# Design and Enzyme-Targeted Assessment of 1,2,4-Triazole–1,8-Naphthalimide Hybrids in Drug Discovery

**DOI:** 10.1155/bmri/6115993

**Published:** 2025-08-10

**Authors:** Nataliya Korol, Mykhailo Slyvka, Ivan Rusyn, Oleksandra Pallah, Svitlana Burmei, Viktoriia Bestritska

**Affiliations:** ^1^Department of Organic Chemistry, Educational and Research Institute of Chemistry and Ecology, Uzhhorod National University, Uzhhorod, Ukraine; ^2^Research Development and Educational Centre of Molecular Microbiology and Mucosal Immunology, Uzhhorod National University, Uzhhorod, Ukraine

## Abstract

This study reports the design, synthesis, and biological evaluation of novel hybrid 1,2,4-triazole–1,8-naphthalimide derivatives using both classical and environmentally friendly synthetic routes. The synthetic strategy involved a multistep process starting with the preparation of triazolylthioacetic acid esters, followed by electrophilic cyclization—employing both conventional bromine and a green, bromine-free method—and culminating in amidation reactions to yield the target compounds. Structural modifications, including the incorporation of pyridinyl and benzoate moieties, were introduced to enhance biological activity. The compounds were evaluated for antimicrobial and anti-inflammatory activities. In antimicrobial assays, several derivatives demonstrated selective activity, with Compound *5f* showing the strongest broad-spectrum antibacterial effects, particularly against *Bacillus cereus* and *Lactobacillus plantarum*, and Compound *3e* displaying notable dual-action activity against both bacterial and fungal strains. These observations underscore the influence of specific functional groups on microbial targeting and membrane penetration. Anti-inflammatory potential was assessed via IL-6 inhibition using ELISA. Significant reductions in IL-6 levels were observed for Compounds *3a*, *3c*, *3e*, *4*, *5c*, *5f*, and *5g*, indicating promising activity, with Compounds *5c* and *3a* reducing IL-6 levels to 7 pg/mL. The complementary molecular docking studies revealed strong binding affinities for Compound *5f* across multiple bacterial enzymes, suggesting effective interactions through hydrogen bonding, electrostatic, and hydrophobic forces and supporting its potential to target iron-regulated pathways and fosfomycin resistance mechanisms. Overall, the integrative approach combining synthetic chemistry, biological assays, and computational modeling highlights the potential of these hybrid 1,2,4-triazole derivatives as candidates for further development as antimicrobial and anti-inflammatory agents.

## 1. Introduction

The chemistry of triazoles is highly diverse and holds considerable significance due to their wide array of valuable properties. These compounds have found extensive applications in biological and medicinal fields [1–7]. Functional and condensed triazoles have become a cornerstone in modern organic and medicinal chemistry. Their biological relevance is particularly evident in their antimicrobial, anti-inflammatory, anticancer, and antiviral activities, which make them invaluable tools for drug development [8–13]. In agriculture, 1,2,4-triazoles are widely used as fungicides and plant growth regulators, playing a crucial role in ensuring sustainable crop productivity [14–17]. Furthermore, their potential as corrosion inhibitors [18–21] and hybride perovskite materials [22] highlights their significance in industrial applications. In addition, triazole's derivatives are well-known functional materials due to catalytic applications [23, 24], chemosensors for sensing applications [25], and polymer memory applications [26]. Their unique structural features, which include remarkable stability and versatile chemical reactivity, make them a subject of great interest for researchers across various disciplines. This broad versatility emphasizes the importance of developing new derivatives of triazoles, both to explore novel applications and to improve existing ones.

Recent studies have continued to highlight the pharmacological importance of 1,2,4-triazole derivatives and their hybrid analogs. For example, Chabhadiya et al. [27] demonstrated enhanced antibacterial and antifungal properties of quinoline–1,2,3-triazole hybrids, particularly against *Staphylococcus aureus* and *Candida albicans* [27], while Rana et al. [28] synthesized naphthalimide–coumarin hybrids exhibiting strong antibacterial activity against multidrug-resistant *Staphylococcus aureus* and *Mycobacterium tuberculosis* [28]. Additionally, green synthetic approaches for heterocyclic scaffolds, including halogen-free oxidative cyclizations, have gained momentum due to sustainability concerns [29]. Computational modeling continues to be a powerful complement to in vitro testing, as recent docking studies have successfully predicted biofilm inhibition potential in triazole-based agents [30].

Our research group has long been dedicated to investigating the synthesis of fused 1,2,4-triazoles and studying their biological activities [31–37]. This work has significantly advanced our understanding of the potential of these compounds and their structural variations. Fused triazole derivatives often exhibit enhanced biological properties, owing to their extended conjugation and increased interaction potential with biological targets, making them promising candidates for further exploration.

**Figure 1:**
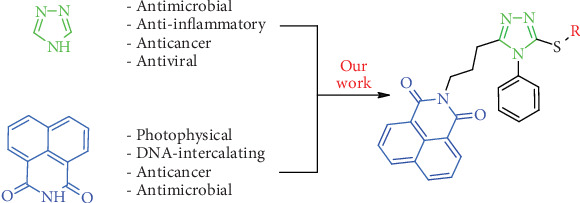
Schematic representation of hybrid 1,2,4-triazole–1,8-naphthalimide compounds, illustrating their origins and key biological properties.

In the present study, we aim to expand the chemical diversity of 1,2,4-triazoles by incorporating another intriguing heterocyclic scaffold—1,8-naphthalimide (Figure 1). This moiety has attracted considerable attention due to its photophysical properties, DNA-intercalating ability, and proven anticancer and antimicrobial activities [38–44]. By merging these two bioactive heterocycles, we seek to create a novel series of hybrid compounds with potentially synergistic properties.

The primary objectives of this study are to synthesize hybrid 1,2,4-triazole–1,8-naphthalimide derivatives and evaluate their antibacterial and anti-inflammatory activities. Additionally, molecular docking studies will be employed to investigate their binding interactions with biological targets, offering valuable insights into their mechanisms of action. This integrative approach, combining synthetic chemistry, biological evaluation, and computational modeling, aims to highlight the potential of these hybrid compounds as therapeutic agents and functional materials.

## 2. Materials and Methods

### 2.1. Chemistry

All reagents were obtained from commercial suppliers and used without any further purification. The melting points were determined on a Stuart SMP30 instrument. Hydrogen-1 NMR (400 MHz) and carbon-13 NMR (100 MHz) spectra were recorded in (CD_3_)_2_SO as a solvent and TMS as an internal standard on a Varian VXR 400. Elemental analyses were performed using an Elementar Vario MICRO cube analyzer, and all experimental results were in full agreement with the calculated values. The NMR spectra (^1^H and ^13^C) for all synthesized compounds are provided in File S1.

#### 2.1.1. Synthesis of Compound 1

The synthesis of triazole *1* was performed according to the reaction between carboxylic acid hydrazide and isothiocyanate as described in the literature [45].

4-Phenyl-5-[2-propyl-1H-benzo[de]isoquinoline-1,3(2*H*)-dione]-1,2,4-triazole-3-thione *1* is as follows: It is a white powder, mp = 235°C, yielding 92%; ^1^H NMR *δ*: 1.93 (m, 2H); 2.47 (t, 2H, *J* = 7.2 Hz), 4.03 (t, 2H, *J* = 6.8 Hz), 7.38 (m, 5H), 7.83 (t, 2H, *J* = 7.6 Hz), 8.40 (d, 2H, *J* = 8.2 Hz), 8.42 (d, 2H, *J* = 8.2 Hz), 13.62 (s, 1H). ^13^C NMR *δ*: 23.06, 23.54, 38.52, 121.90, 127.08, 127.29, 128.19, 129.16, 130.61, 131.18, 133.62, 134.20, 151.60, 163.38, 167.66.

#### 2.1.2. Synthesis of 3a–3e

The synthesis of esters of triazolylthioacetic acid was achieved using an alkylation reaction. This reaction followed an analogous technique previously described in the literature [46].

Potassium 4-phenyl-5-[2-propyl-1H-benzo[de]isoquinoline-1,3(2*H*)-dione]-4*H*-1,2,4-triazole-3-thiolate *3a* is as follows: It is a yellow powder, mp = 255°C, yielding 90%; ^1^H NMR *δ*: 1.82 (m, 2H); 2.42 (t, 2H, *J* = 7.3 Hz), 3.99 (t, 2H, *J* = 6.9 Hz), 7.18 (m, 3H), 7.28 (t, 2H, *J* = 7.5 Hz), 7.84 (t, 2H, *J* = 7.6 Hz), 8.42 (m, 4H). ^13^C NMR *δ*: 21.95, 23.57, 24.90, 39.03, 121.95, 125.59, 127.08, 128.16, 128.39, 130.63, 131.20, 134.18, 136.49, 149.93, 163.23, 166.75.

4-Phenyl-5-[2-propyl-1H-benzo[de]isoquinoline-1,3(2*H*)-dione]-3-[(3-phenylprop-2-en-1-yl)sulfanyl]-4*H*-1,2,4-triazole *3b* is as follows: It is a white powder, mp = 164°C, yielding 87%; ^1^H NMR *δ*: 1.96 (m, 2H); 2.58 (t, 2H, *J* = 7.2 Hz), 3.81 (d, 2H, *J* = 5.4 Hz), 4.05 (t, 2H, *J* = 6.8 Hz), 6.25 (m, 1H), 6.51 (d, 1H, *J* = 10.2 Hz), 7.20 (t, 1H, *J* = 7.5 Hz), 7.29 (t, 2H, *J* = 7.5 Hz), 7.37 (d, 2H, *J* = 8.0 Hz), 7.43 (m, 5H), 7.86 (m, 2H), 8.45 (m, 4H). ^13^C NMR *δ*: 22.56, 24.56, 35.10, 38.84, 122.01, 124.55, 126.22, 127.12, 127.28, 127.69, 128.55, 129.61, 130.65, 130.78, 131.24, 132.86, 133.08, 134.22, 136.11, 140.54, 149.02, 155.04, 163.39.

Ethyl [(5-[2-propyl-1*H*-benzo[de]isoquinoline-1,3(2*H*)-dione]-4-phenyl-4*H*-1,2,4-triazol-3-yl)sulfanyl]acetate *3c* is as follows: It is a white powder, mp = 133°C, yielding 94%; ^1^H NMR *δ*: 1.16 (t, 3H, *J* = 7.1 Hz), 1.96 (m, 2H), 2.60 (t, 2H, *J* = 7.3 Hz), 3.95 (s, 2H), 4.06 (m, 4H), 7.44 (m, 5H), 7.82 (t, 2H, *J* = 7.6 Hz), 8.41 (m, 4H).

Ethyl 4-[(5-[2-propyl-1*H*-benzo[de]isoquinoline-1,3(2*H*)-dione]-4-phenyl-4*H*-1,2,4-triazol-3-yl)sulfanyl]butanoate *3d* is as follows: It is a white powder, mp = 65°C, yielding 90%; ^1^H NMR *δ*: 1.15 (t, 3H, *J* = 7.1 Hz), 1.82 (m, 2H), 1.98 (m, 2H); 2.32 (t, 2H, *J* = 7.4 Hz), 2.59 (t, 2H, *J* = 7.3 Hz), 2.99 (t, 2H, *J* = 6.9 Hz), 4.04 (m, 4H), 7.42 (m, 5H), 7.86 (t, 2H, *J* = 7.6 Hz), 8.47 (m, 4H). ^13^C NMR *δ*: 14.02, 22.55, 24.35, 24.46, 31.40, 32.07, 39.02, 59.80, 122.00, 127.11, 127.23, 127.37, 129.64, 130.63, 131.24, 133.03, 134.21, 136.67, 149.29, 154.94, 163.41, 172.16.

Methyl 4-{[(5-[2-propyl-1*H*-benzo[de]isoquinoline-1,3(2*H*)-dione]-4-phenyl-4*H*-1,2,4-triazol-3-yl)sulfanyl]methyl}benzoate *3e* is as follows: It is a white powder, mp = 170°C, yielding 89%; ^1^H NMR *δ*: 1.95 (m, 2H); 2.56 (t, 2H, *J* = 7.3 Hz); 3.80 (s, 3H), 4.01 (t, 2H, *J* = 6.8 Hz), 4.30 (s, 2H), 7.27 (d, 2H, *J* = 8.1 Hz), 7.39 (m, 5H), 7.82 (m, 4H), 8.39 (m, 4H).

#### 2.1.3. Synthesis of 4

The synthesis of Tribromode *4* was performed according to the following synthetic routes:

1. One millimole of cinnamyl thioether 3b was dissolved in 10 mL of glacial acetic acid, and 2 mmol of the bromine in 5 mL of glacial acetic acid was dropped. The reaction mixture was stirred at room temperature for 24 h. The cyclization product was isolated by filtration and purified by crystallization in DMF‐AcOH = 1 : 2.
2. One millimole of cinnamyl thioether 3b was dissolved in 10 mL of acetonitrile and 2 mmol of NaBr, and 4 mmol of K_2_S_2_O_8_ was added. The reaction mixture was stirred at room temperature for 48 h. The cyclization product was isolated from the filtrate and purified by crystallization in DMF‐AcOH = 1 : 2.

6-Bromo-2-[2-propyl-1*H*-benzo[de]isoquinoline-1,3(2*H-*)dione]-3,7-diphenyl-3,5,6,7-tetrahydro[1,2,4]triazolo[5,1-*b*][1,3]thiazin-8-ium tribromide *4* is as follows: It is an off-white powder, mp = 182°C, yielding 89%^1^; 52%^2^; ^1^H NMR *δ*: 2.07 (m, 2H); 2.63 (t, 2H, *J* = 7.2 Hz); 3.44 (s, 1H); 3.75 (d, 2H, *J* = 5.4 Hz); 4.02 (t, 2H, *J* = 6.8 Hz); 5.44 (s, 1H); 6.24 (m, 1H); 7.42 (t, 1H, *J* = 7.5 Hz); 7.64 (t, 2H, *J* = 7.5 Hz); 7.80 (d, 2H, *J* = 8.0 Hz); 7.86 (m, 5H); 7.88 (m, 2H); 8.45 (m, 4H).

#### 2.1.4. Synthesis of 5a–5g

The synthesis of Amines *5a*–*5g* was performed according to the synthetic routes:

1. Two millimoles of ester *3c* was dissolved in 2 mL of DMF, and 10 mmol of the amine was added. The reaction mixture was boiled on a water bath for 2 h. The solvent was evaporated under vacuum (20 mmHg), and the dry residue was washed with three portions of 5 mL of distilled water. The isolated amide was recrystallized from ethanol or a mixture: ethanol‐DMF = 3 : 1.
2. Two millimoles of ester *3c* was dissolved in 2 mL of the corresponding amine when heated. The reaction mixture was kept at a temperature of 100–120° for 10 min and left for 24 h. Upon cooling, the target product precipitated, which was filtered, washed with three portions of 5 mL of distilled water. The isolated amide was recrystallized from ethanol or a mixture: ethanol‐DMF = 3 : 1.

N-[3-(3,5-Dimethoxyphenyl)propyl]-2-[(4-phenyl-5-[2-propyl-1*H*-benzo[de]isoquinoline-1,3(2*H*)-dione]-4*H*-1,2,4-triazol-3-yl)sulfanyl]acetamide *5a* is as follows: It is an off-white powder, mp = 182°C, yielding 72%^1^; ^1^H NMR *δ*: 1.97 (m, 2H), 2.61 (m, 4H), 3.23 (m, 2H), 3.70 (s, 6H), 3.80 (s, 2H), 4.06 (t, 2H, *J* = 6.5 Hz), 6.68 (d, 1H, *J* = 2.0 Hz), 6.78 (s, 1H), 6.82 (d, 1H, *J* = 2.0 Hz), 7.44 (m, 5H), 7.87 (t, 2H, *J* = 9.0 Hz), 8.22 (t, 1H, *J* = 7.5 Hz), 8.45 (d, 4H, *J* = 6.0 Hz). ^13^C NMR *δ*: 22.43, 29.40, 32.94, 34.42, 43.29, 55.49, 56.17, 111.91, 112.55, 120.39, 127.13, 127.38, 129.70, 130.66, 131.24, 132.86, 134.23, 148.59, 149.48, 154.90, 163.41.

2-[(4-Phenyl-5-[2-propyl-1*H*-benzo[de]isoquinoline-1,3(2*H*)-dione]-4*H*-1,2,4-triazol-3-yl)sulfanyl]-*N*-[(furan-2-yl)methyl]acetamide *5b* is as follows: It is a white powder, mp = 182°C, yielding 69%^1^; ^1^H NMR *δ*: 1.97 (m, 2H), 2.60 (t, 2H, *J* = 9.0 Hz), 3.86 (s, 2H), 4.06 (t, 2H, *J* = 9.0 Hz), 4.24 (d, 2H, *J* = 5.0 Hz), 6.23 (d, 1H, *J* = 3.5 Hz), 6.37 (dd, 1H, *J* = 3.5, 1.5 Hz), 7.41 (m, 5H), 7.55 (d, 1H, *J* = 8.0 Hz), 6.86 (s, 1H), 7.15 (s, 1H), 7.43 (m, 5H), 7.82 (t, 2H, *J* = 7.5 Hz), 7.87 (t, 2H, *J* = 7.5 Hz), 8.46 (d, 4H, *J* = 6.0 Hz), 8.65 (t, 1H, *J* = 5.5 Hz).

2-[(4-Phenyl-5-[2-propyl-1*H*-benzo[de]isoquinoline-1,3(2*H*)-dione]-4*H*-1,2,4-triazol-3-yl)sulfanyl]-*N*-[3-(morpholin-4-yl)propyl]acetamide *5c* is as follows: It is an off-white powder, mp = 103°C, yielding 65%^1^, 63%^2^; ^1^H NMR *δ*: 1.51 (m, 2H), 1.96 (m, 2H), 2.23 (t, 2H, *J* = 7 Hz), 2.29 (m, 4H), 2.60 (t, 2H, *J* = 9 Hz), 3.06 (dd, 2H, *J* = 6, 12 Hz), 3.53 (m, 4H), 3.72 (s, 2H), 4.06 (t, 2H, *J* = 9 Hz), 7.44 (m, 5H), 7.87 (t, 2H, *J* = 8 Hz), 8.16 (t, 1H, *J* = 7 Hz), 8.46 (dd, 4H, *J* = 6, 9 Hz). ^13^C NMR *δ*: 22.43, 25.69, 28.31, 33.14, 38.17, 38.71, 46.55, 53.24, 66.14, 122.49, 127.13, 129.70, 130.66, 132.67, 134.24, 149.26, 159.60, 163.41, 172.27.

2-[(4-Phenyl-5-[2-propyl-1*H*-benzo[de]isoquinoline-1,3(2*H*)-dione]-4*H*-1,2,4-triazol-3-yl)sulfanyl]-1-[4-(2-hydroxyethyl)piperazin-1-yl]ethan-1-one *5d* is as follows: It is an off-white powder, mp = 177°C, yielding 65%^1^; ^1^H NMR *δ*: 1.97 (m, 2H), 2.33 (t, 2H, *J* = 7 Hz), 2.38 (m, 2H), 2.60 (t, 2H, *J* = 9 Hz), 3.39 (m, 4H), 3.49 (dd, 2H, *J* = 6, 12 Hz), 4.05 (t, 2H, *J* = 8 Hz), 4.12 (s, 2H), 4.41 (t, 1H, *J* = 5 Hz), 7.44 (m, 5H), 7.86 (t, 2H, *J* = 8 Hz), 8.45 (d, 4H, *J* = 7 Hz).

2-[(4-Phenyl-5-[2-propyl-1*H*-benzo[de]isoquinoline-1,3(2*H*)-dione]-4*H*-1,2,4-triazol-3-yl)sulfanyl]-*N*-[3-(1*H*-imidazol-1-yl)propyl]acetamide *5e* is as follows: It is a white powder, mp = 160°C, yielding 76%^1^, 71%^2^; ^1^H NMR *δ*: 1.81 (m, 2H), 1.96 (m, 2H), 1.97 (m, 2H), 2.60 (t, 2H, *J* = 9 Hz), 3.01 (m, 2H), 3.82 (s, 2H), 3.95 (t, 2H, *J* = 9 Hz), 4.03 (t, 4H, *J* = 9 Hz), 6.86 (s, 1H), 7.15 (s, 1H), 7.43 (m, 5H), 7.82 (t, 2H, *J* = 8 Hz), 8.25 (t, 1H, *J* = 7 Hz), 8.37 (d, 4H, *J* = 6 Hz). ^13^C NMR *δ*: 22.43, 24.61, 30.54, 35.87, 36.05, 38.82, 40.03, 43.40, 119.26, 121.90, 127.05, 127.14, 128.26, 129.70, 130.59, 131.16, 132.86, 134.16, 137.24, 149.49, 154.96, 163.33, 166.75.

2-[(4-Phenyl-5-[2-propyl-1*H*-benzo[de]isoquinoline-1,3(2*H*)-dione]-4*H*-1,2,4-triazol-3-yl)sulfanyl]-*N*-[(pyridin-3-yl)methyl]acetamide *5f* is as follows: It is a white powder, mp = 181°C, yielding 68%^2^; ^1^H NMR *δ*: 1.97 (m, 2H), 2.60 (t, 2H, *J* = 9 Hz), 3.89 (s, 2H), 4.06 (t, 2H, *J* = 8 Hz), 4.29 (d, 2H, *J* = 6 Hz), 7.32 (dd, 1H, *J* = 4, 8 Hz), 7.43 (m, 5H), 7.63 (d, 1H, *J* = 6 Hz), 7.87 (t, 2H, *J* = 9 Hz), 8.46 (m, 6H), 8.73 (t, 1H, *J* = 5 Hz). ^13^C NMR *δ*: 22.95, 25.14, 35.87, 39.38, 56.69, 122.52, 123.85, 125.77, 127.64, 127.88, 130.20, 131.17, 133.01, 133.35, 134.52, 134.74, 134.94, 135.44, 137.82, 148.54, 149.11, 163.73, 163.74, 165.27, 165.29, 167.44.

2-[(4-Phenyl-5-[2-propyl-1*H*-benzo[de]isoquinoline-1,3(2*H*)-dione]-4*H*-1,2,4-triazol-3-yl)sulfanyl]-1-(piperidin-1-yl)ethan-1-one *5g* is as follows: It is a white powder, mp = 176°C, yielding 74%^1^; ^1^H NMR *δ*: 1.39 (m, 2H), 1.52 (m, 4H), 1.97 (m, 2H), 2.60 (m, 2H, *J* = 9 Hz), 3.35 (m, 4H), 4.05 (m, 4H, *J* = 8 Hz), 4.11 (m, 4H, *J* = 7 Hz), 7.44 (m, 5H), 7.85 (t, 2H, *J* = 9 Hz), 8.44 (d, 4H, *J* = 6 Hz). ^13^C NMR *δ*: 22.49, 23.72, 24.58, 25.07, 25.72, 36.44, 42.37, 121.99, 126.98, 127.10, 127.19, 127.36, 129.68, 130.62, 131.22, 132.98, 134.18, 149.40, 154.82, 163.30, 164.60.

### 2.2. Biological Activity

#### 2.2.1. Antimicrobial Activity

The study was conducted in the laboratory of the Research and Training Center for Molecular Microbiology and Immunology of Mucous Membranes, State Higher Educational Institution, Uzhhorod National University. The antagonistic activity of the provided organic compounds was assessed through cocultivation experiments. The test subjects included clinical isolates of opportunistic microorganisms *Klebsiella oxytoca KS02*, *Pseudomonas aeruginosa KS12*, *Serratia ficaria KS22* (Gram-negative rods), and *Staphylococcus aureus KS32* (Gram-positive cocci) and microscopic fungi *Candida albicans KSF11* and *Geotrichum candidum KSF22*. Additionally, the impact on commensal microorganisms was evaluated, including *Escherichia coli KS43* (lac+), *Enterococcus faecalis KS54*, and the probiotic strain *Lactobacillus plantarum* A. The test strain *Bacillus cereus* ATCC 11778 from the Ukrainian Collection of Microorganisms was also included. In total, 10 microorganisms were used in the experiments. The antagonistic activity of 13 organic preparations and the solvent was quantitatively analyzed [47]. Suspensions of microorganisms with an optical density of 0.5 McFarland (corresponding to 1.5 × 10^8^ CFU/mL) were prepared from 24-h bacterial cultures. The purity and morphological characteristics of the test microorganisms were verified using bacterioscopy (Gram staining) and light microscopy (Primo Star iLED, Carl Zeiss). In the experiments, 10 *μ*L of the prepared bacterial suspensions and 100 *μ*L of each preparation were added to sterile 96-well plates. Controls included microorganism suspensions without preparations and preparations without bacterial suspensions. Plates were incubated at 37°C for 2 h, followed by titration using the serial dilution method and subsequent inoculation of all tested strains onto Petri dishes with appropriate nutrient media (MPA, Sabouraud, MRS, Endo, Enterococcal). Results were evaluated after 24–48 h, depending on the type of microorganism. The sterility of the organic compounds was assessed bacteriologically by inoculating 10 *μ*L of each preparation onto meat-peptone agar, a universal nutrient medium for cultivating microorganisms. The sterility tests confirmed that all provided compound samples were free from microbial contamination. Three types of control groups were used: (1) microbial growth control—bacterial suspension without compounds to confirm viability; (2) compound sterility control—compounds without microorganisms, plated on nutrient media to exclude contamination; and (3) solvent control—solvent alone at the working concentration to exclude its antimicrobial effect. These controls ensured reliable interpretation of the observed antimicrobial activity. The antimicrobial activity of the synthesized compounds was evaluated using a modified microbroth cocultivation approach in 96-well plates, followed by serial dilution and viable cell counting on selective media. While standard antibiotics were not included in the assay design, the general methodological framework was adapted in accordance with international guidelines such as the Clinical and Laboratory Standards Institute (CLSI) M07 (https://clsi.org/shop/standards/m07/) and the EUCAST principles for in vitro antimicrobial testing (https://www.eucast.org/). These procedures were employed to assess the direct inhibitory effect of the compounds on microbial growth rather than to determine classical minimum inhibitory concentrations (MICs) or resistance profiles. The method was specifically optimized for novel organic compounds and allowed for comparative evaluation of their antimicrobial potential across a spectrum of clinical and reference strains.

#### 2.2.2. Anti-Inflammatory Activity

The IL-6 level in blood serum was determined using the Human IL-6 ELISA Kit (Elabscience, United States) according to the manufacturer's instructions. The IL-6 determination method was based on that described by Hwang et al. [48]. Briefly, biological serum samples provided by the Research and Educational Center for Molecular Microbiology and Mucosal Immunology of the State Higher Educational Institution “Uzhhorod National University” were centrifuged to obtain the supernatant. The obtained samples were stored at −80°C until analysis. Before conducting the ELISA, the samples were brought to room temperature and then added to the wells of a 96-well plate according to the manufacturer's instructions. Incubation and washing were performed according to the standard ELISA protocol [49]. Optical density was measured using a microplate reader at 450 nm (BioTeK, United States). The analysis results were processed using a nonlinear regression method to determine the percentage of IL-6 inhibition relative to control samples [48].

### 2.3. Molecular Docking

Molecular docking studies of Compounds 1 and 2 that were docked into the crystal structures of proteins with PDB_ID: 8AVI, 8DTD, 3LP5, 4C87, 1DHI, 1T2P, 4ZZT, and 7STO were carried out using AutoDock Vina software [50], an open-source molecular docking software. Firstly, we optimized the structures of enzymes in BIOVIA Discovery Studio Visualizer 2021 [51]. This optimization process included creating the grid box for each protein, adding charges, and refining the structures. The structures of compounds were prepared by Avogadro [52]. We generated 10 conformations in each docking output by using the advanced genetic algorithm method in Vina Protein/TP. The molecule input preparations and docking output analysis and visualization were carried out using Discovery Studio software.

## 3. Results and Discussion

### 3.1. Synthesis

**Scheme 1:**
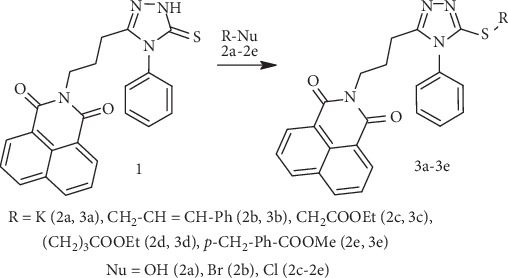
Synthesis of alkylated triazole derivatives 3a–3e via alkylation of Compound 1.

The synthesis of the target compounds was carried out through a multistep process, ensuring precision and efficiency at each stage. The first step involved the synthesis of esters *3a*–*3e* of triazolylthioacetic acid, achieved using an alkylation reaction from triazole *1* (Scheme 1).

**Scheme 2:**
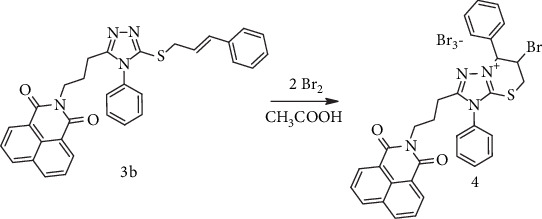
Electrophilic bromocyclization of Compound 3b leading to the formation of Salt 4.

In the second step, thioether *3b* was converted into triazolium tribromide *4* (Scheme 2) via a classical electrophilic cyclization approach (the action of bromine) and via an alternative green technique without the usage of bromine. The first route of this conversion was achieved by reacting the cinnamyl ester with a double molar excess of bromine in acetic acid, following the standard electrophilic heterocyclization protocol. The second path was performed with the aim of investigating the possibility of implementing green approaches into classical electrophilic heterocyclization methods—namely, carrying out bromoheterocyclization without using toxic bromine as an electrophile. We have used the mixture of NaBr and K_2_S_2_O_8_ in acetonitrile for the generation of Br^+^ electrophile in situ. This approach was successfully used for the halogenation of imidazo-fused heterocycles [53].

**Scheme 3:**
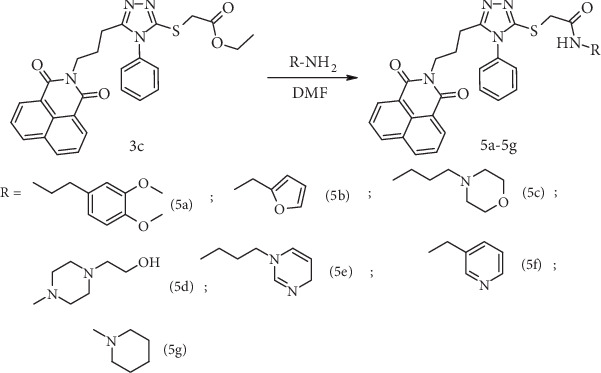
Synthesis of amides 5a–5g from ester 3c via aminolysis.

The final stage of the synthetic route, aimed at obtaining the target compounds, involved the preparation of the corresponding amides *5a*–*5g* from the ester *3c*. This transformation was performed by reacting the esters with the appropriate amines in the presence of dimethylformamide in DMF, as well as via a green solvent-free procedure (Scheme 3).

This stepwise approach highlights the strategic planning involved in synthesizing the target compounds, demonstrating the utility of well-established organic reactions to achieve structural complexity. All the obtained compounds were tested for their biological and anti-inflammatory activities, and the study was finalized with a molecular docking evaluation.

### 3.2. Biological Activity

The synthesized series of 1,2,4-triazole derivatives was evaluated for their antimicrobial potential by exposing various pathogenic and opportunistic microorganisms—including both Gram-negative and Gram-positive bacteria as well as fungi—to these compounds at specified concentrations. The study measured changes in colony forming units per milliliter to assess efficacy, revealing that while most compounds showed limited activity, certain candidates such as *5f*, *3e*, and *4* significantly reduced microbial counts. All the measured results are summarized in Table 1.

Among the tested microorganisms, the *Bacillus cereus* ATCC 11778 strain exhibited the highest resistance to the compounds. *Bacillus cereus* is a Gram-positive, facultatively anaerobic, motile, spore-forming rod-shaped bacterium widely distributed across diverse environments, including soil, plants, water, and the intestinal tract. The results revealed that the studied compounds were generally more effective against Gram-negative bacteria (*Escherichia coli (lac+)*, *Klebsiella oxytoca*, *Pseudomonas aeruginosa*, and *Serratia ficaria*) compared to Gram-positive bacteria. These findings underscore the potential of the studied compounds as promising antimicrobial agents, particularly against Gram-negative bacteria, and highlight the need for further studies to elucidate their mechanisms of action, safety profiles, and efficacy across a broader range of pathogenic microorganisms.

The most effective compounds were those that significantly reduced microbial concentrations compared to the control. Among these, Compound *5f* demonstrated the strongest activity, substantially reducing *Bacillus cereus* concentrations to 1 · 10^6^, the most significant reduction observed. Additionally, *5f* was effective against *Lactobacillus plantarum A*, reducing concentrations to 1 · 10^8^, indicating broad-spectrum antimicrobial potential. Compound *3e* was also noteworthy, reducing *Bacillus cereus* to 1 · 10^8^ and demonstrating activity against *Staphylococcus aureus* (1 · 10^6^) and *Geotrichum candidum* (2.4 · 10^7^). These results suggest that *3e* has broad-spectrum activity and efficacy against clinically significant pathogens. Compound *4* showed promising activity by reducing *Bacillus cereus* concentrations to 3 · 10^7^, though its activity appeared more selective.

In contrast, most of the other tested compounds (*1*, *3a*–*3d*, and *5a*–*5g*) did not show measurable reductions in microbial concentrations across the tested strains, indicating limited antimicrobial activity. This lack of activity can be attributed to factors such as low lipophilicity, nonselective binding, lipophilicity–hydrophilicity imbalance, or the absence of key reactive functional groups necessary for effective microbial targeting.

The observed biological activities of these compounds highlight the impact of structural modifications in 1,2,4-triazole derivatives on their antimicrobial potency. For instance, Compound *5f*'s strong broad-spectrum activity may be attributed to the presence of a pyridinyl moiety at the third position of the 1,2,4-triazole core. Pyridinyl moieties are associated with high electron density, facilitating binding to nucleophilic enzyme pockets [54, 55]. The pyridine ring likely enhances activity by improving hydrophilicity and electronic interactions [56, 57], enabling better binding to microbial targets.

Compound *3e* demonstrated efficiency against multiple pathogens, including the pathogenic fungus *Geotrichum candidum* and Gram-positive bacterium *Staphylococcus aureus*, suggesting utility as a dual-action antimicrobial and antifungal agent. The benzoate group in *3e* likely targets bacterial esterases [58] or similar enzymes, disrupting metabolic processes. Additionally, the benzoate ester increases lipophilicity [59], enhancing membrane penetration in both bacterial and fungal cells and improving access to intracellular targets.

The varying biological activities of the tested compounds emphasize the critical role of chemical structure in determining antimicrobial efficacy. Compounds *5f* and *3e* stood out due to their favorable functional group profiles, which enhance interactions with microbial targets. Conversely, less active compounds suffered from structural limitations, including steric hindrance, suboptimal lipophilicity–hydrophilicity balance, or absence of key reactive groups.

The structure–activity relationship (SAR) analysis reveals that specific functional groups contribute markedly to the observed biological activities. Notably, Compound *5f*, bearing a pyridinyl moiety, demonstrated both strong antimicrobial and anti-inflammatory properties. Pyridinyl rings are known to improve aqueous solubility and enhance binding to nucleophilic sites within enzyme pockets due to their electron-deficient nature [60]. This facilitates hydrogen bonding and *π*–*π* stacking interactions, as observed in docking studies with IsdG and FosB enzymes.

Similarly, Compound *3e*, which includes a benzoate ester, exhibited dual antibacterial and antifungal activity. The ester increases lipophilicity, potentially enhancing membrane penetration in microbial cells. Furthermore, benzoate esters are often substrates for bacterial esterases, leading to selective intracellular activation and inhibition [61]. These effects have been described in ester-linked prodrugs targeting Gram-positive bacteria.

These observations align with recent studies on 1,2,4-triazole hybrids where aryl and heteroaryl substituents influenced microbial selectivity through changes in polarity, electron density, and steric hindrance [62].

These findings warrant further investigations into the mechanisms of action of these compounds, alongside studies to evaluate their toxicity, pharmacokinetics, and potential therapeutic applications. The data underscore the importance of targeted structural modifications in developing effective antimicrobial agents.

### 3.3. Anti-Inflammatory Activity

For investigating the anti-inflammatory activity of the compounds, we conducted a concise analysis of the ELISA data to assess their potential to reduce IL-6 levels relative to a control sample. IL-6, a key proinflammatory cytokine, serves as a marker of the inflammatory response, and its reduction indicates potential therapeutic efficacy. All results are summarized in Table 2.

The results of the ELISA provide valuable insights into the anti-inflammatory potential of the synthesized compounds, evaluated by their ability to reduce IL-6 levels. IL-6 is a crucial proinflammatory cytokine implicated in various diseases, including rheumatoid arthritis, inflammatory bowel disease, and certain cancers [63–65]. A reduction in IL-6 levels indicates potential therapeutic efficacy, and the results of this study reveal a diverse range of activities among the compounds, offering a basis for SAR analysis.

The control group, which contained no compounds, maintained a consistent IL-6 level of 12 pg/mL, serving as a baseline for activity assessment. Compounds that reduced IL-6 levels below this threshold were classified as active. Among the tested compounds, *5c* and *3a* demonstrated the most significant activity, reducing IL-6 levels to 7 pg/mL, highlighting their strong potential as anti-inflammatory agents. These compounds likely feature structural elements that enhance their interaction with inflammatory pathways, such as balanced hydrophilic and hydrophobic properties or strategically positioned functional groups like sulfanyl and ester moieties, which may contribute to enhanced target affinity.

Compounds *4*, *5f*, *3c*, *5g*, and *3e* reduced IL-6 levels to 8 pg/mL, exhibiting consistent and noteworthy activity. The activity of these compounds can be attributed to their functional groups; for instance, the pyridinyl group in *5f* not only enhances its anti-inflammatory properties [66, 67] but may also align with its previously noted antimicrobial efficacy, suggesting dual activity. Similarly, the benzoate ester group in *3e* may facilitate membrane penetration and effective interaction with inflammatory targets [68, 69], contributing to its moderate activity.

A further group of compounds, including *5e*, *5d*, *3d*, and *5a*, demonstrated mild activity by reducing IL-6 levels to 9–10 pg/mL. These compounds may possess amide or heterocyclic groups that enable some degree of interaction with inflammatory targets, although steric hindrance or insufficient reactivity might limit their overall efficacy. These compounds present opportunities for optimization to improve their bioactivity.

In contrast, Compounds *5b*, *3b*, and *1* were inactive, failing to significantly reduce IL-6 levels, with their values remaining at or near the control level of 12 pg/mL. Compound *5b*, despite containing a furan ring, likely suffers from reduced hydrophobicity and limited electronic interactions, which could impair its ability to modulate inflammatory pathways. The high hydrophilicity of *3b*'s substituents may hinder its membrane penetration and subsequent interaction with key targets. Compound *1*, featuring a thione group, appears to lack sufficient reactivity to influence IL-6 levels meaningfully.

These findings underscore the critical role of chemical structure in determining bioactivity. Compounds *5c* and *3a*, which exhibited the most significant reduction in IL-6 levels, emerge as promising leads for further pharmacological evaluation. Their structural features warrant detailed exploration to elucidate their mechanisms of action. The moderately active compounds, such as *4*, *5f*, *3c*, *5g*, and *3e*, represent candidates for structural optimization, with modifications aimed at enhancing their potency. On the other hand, the inactive compounds provide valuable insights into structural deficiencies that can guide future design strategies, such as introducing reactive functional groups or reducing steric hindrance to improve target interactions.

In assessing anti-inflammatory activity, Compound *5f* was of particular interest due to its potent IL-6 inhibition and high affinity toward docking targets associated with inflammatory pathways. The pyridinyl moiety in *5f*, widely recognized in medicinal chemistry for mimicking biologically active heterocycles, may enhance interactions with cytokine-modulating proteins, such as TLRs or MAPKs [70]. While these specific targets were not modeled here, the presence of multiple polar interaction sites supports a plausible mechanism.

Compounds with lower activity (e.g., *5b* and *3b*) lacked such pharmacophores or bore highly hydrophilic residues, limiting membrane permeability and target engagement. This structure-dependent activity emphasizes the relevance of electron-rich, heteroaromatic functionalities in cytokine inhibition—a trend consistent with several recent reports [71].

Thus, the anti-inflammatory profile correlates with functional group chemistry. Compounds reducing IL-6 levels below 8 pg/mL (e.g., *3a* and *5c*) often feature polar, flexible side chains capable of interacting with cytokine-regulating targets.

Overall, the ability of these compounds to reduce IL-6 levels highlights their potential as anti-inflammatory agents. This study emphasizes the importance of SAR analysis in identifying and optimizing effective therapeutics, paving the way for further in vivo testing and detailed mechanistic studies to advance these compounds as candidates for treating inflammatory and autoimmune diseases.

### 3.4. Molecular Docking

Molecular docking studies were conducted to investigate the interactions of selected compounds with key proteins from various microorganisms. These proteins were chosen based on their roles in bacterial resistance, virulence, and microbial processes, offering insights into the potential therapeutic applications of the compounds under study. The availability of high-resolution structural data for these proteins ensures accurate docking predictions, which are crucial for developing novel therapeutic agents.

In *Bacillus cereus*, two proteins were selected as targets: FosB and IsdG. FosB is a fosfomycin resistance enzyme that chemically modifies and inactivates fosfomycin, an antibiotic targeting bacterial cell wall synthesis [72]. Since fosfomycin resistance poses a growing challenge, inhibitors of FosB could restore the antibiotic's effectiveness. Additionally, IsdG, an iron-regulated surface determinant enzyme, plays a vital role in bacterial virulence by breaking down heme to release iron—a critical nutrient in host environments [73]. Inhibiting IsdG disrupts iron acquisition, thereby weakening bacterial pathogenicity. Together, these targets address both resistance (FosB) and virulence (IsdG) in *Bacillus cereus*, making them integral to docking studies.

For *Staphylococcus aureus*, two critical proteins were selected: Sortase A and dihydrofolate reductase (DHFR). Sortase A is an enzyme responsible for anchoring surface proteins to the bacterial cell wall, a process vital for adhesion, biofilm formation, and tissue infection [74]. Inhibitors of Sortase A have shown promise in impairing bacterial virulence, making it a valuable drug target. On the other hand, DHFR is an enzyme involved in folate metabolism and DNA synthesis. Although it is not directly related to *β*-lactam antibiotic resistance, targeting DHFR can disrupt essential bacterial metabolic processes, providing an alternative antibacterial strategy [75].

In the case of *Lactobacillus plantarum*, two proteins were chosen to evaluate the potential impact of the studied compounds on beneficial gut microbiota: esterase LpEst1 and a putative cell surface hydrolase. Esterase LpEst1 controls key bacterial adaptive mechanisms, including stress tolerance and metabolism [76]. Targeting this enzyme allows for assessing potential side effects of antimicrobial compounds on beneficial bacteria. Meanwhile, the putative cell surface hydrolase hydrolyzes ester bonds on the bacterial surface, influencing adhesion and microbial interactions in the gut [77]. Understanding the effects of compounds on LpEst1 and cell surface hydrolases ensures that the ecological balance of the gut microbiome is not disrupted, highlighting the importance of safety profiles in antimicrobial design.

For *Geotrichum candidum*, the primary target was Cel7A, a cellulase enzyme involved in lignocellulose degradation. Cel7A's role in fungal cell wall degradation makes it a critical target for studying enzyme–inhibitor interactions. The high-resolution structural data (1.56 Å) for Cel7A complexed with thio-linked cellotriose provides a robust model for designing inhibitors against fungal cell wall enzymes [78]. Additionally, other fungal-specific enzymes, such as chitin synthase and lanosterol 14*α*-demethylase, were considered for their roles in fungal cell wall and membrane biosynthesis [79]. These targets hold significant potential for antifungal drug discovery.

This comprehensive approach to selecting protein targets spans pathogenic bacteria (*Bacillus cereus* and *Staphylococcus aureus*), beneficial microbes (*Lactobacillus plantarum*), and fungi (*Geotrichum candidum*). By addressing antimicrobial efficacy, resistance mechanisms, virulence, and the safety profiles of compounds, these studies provide a multidimensional strategy for therapeutic discovery. Each protein target represents a unique avenue for combating infections while minimizing adverse effects on beneficial microorganisms, ensuring a balanced and effective therapeutic approach.

Compounds *5f* and *3e*, identified as the most biologically active, were further analyzed using molecular docking against selected target proteins based on their activity profiles. Specifically, Compound *3e* was docked onto IsdG, FosB, DHFR, Sortase A, Cel7A, and Chitin Synthase 2, while Compound *5f* was docked onto IsdG, FosB, the putative cell surface hydrolase, and esterase LpEst1. All results are summarized in Table 3.

**Figure 2:**
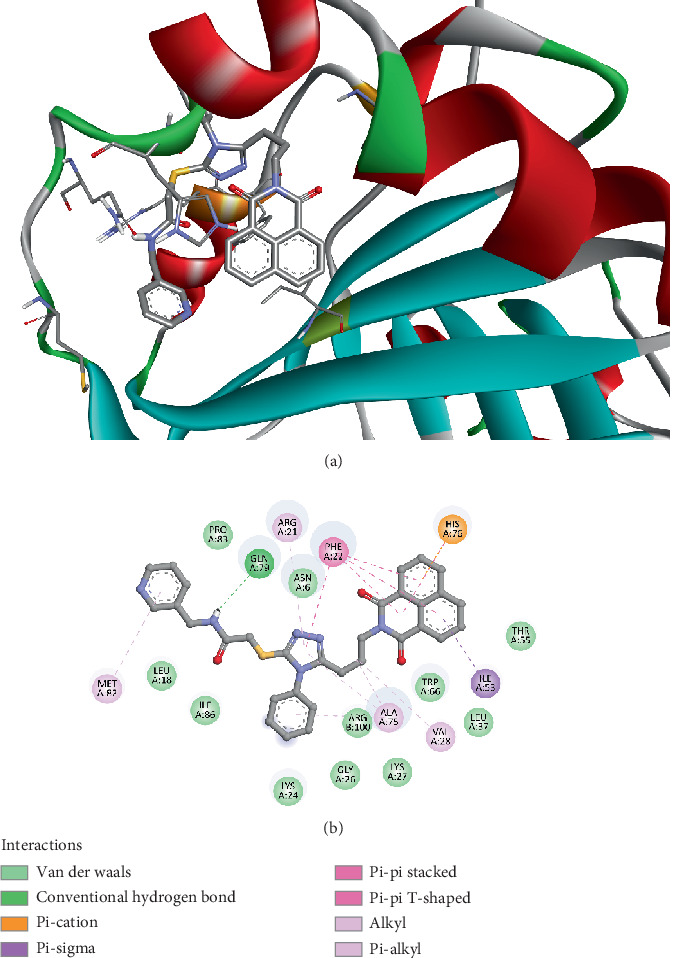
Visualization of molecular docking results showing the interactions between Compound *5f* and the IsdG protein from *Bacillus cereus* (PDB ID: 8AVI) in both (a) 3D and (b) 2D views.

The molecular docking analysis demonstrated that Compound *5f* binds strongly to the IsdG protein from *Bacillus cereus* (PDB ID: 8AVI) with a binding affinity of −10.2 kcal/mol, indicating a high level of stability within the protein's active site. The binding interactions are diverse, involving hydrogen bonding, *π*-stacking, and hydrophobic interactions. Notably, GLN79 forms a conventional hydrogen bond (2.36 Å), contributing to the compound's stability. HIS76 plays a crucial role, engaging in pi–cation and pi–donor hydrogen bonding (2.97 Å), as well as pi–pi stacking interactions (4.85–5.52 Å), which enhance electronic stabilization. Additionally, PHE22 participates in pi–pi T-shaped interactions (4.72–5.26 Å), further strengthening the aromatic stacking effects. ILE53 contributes through a pi–sigma interaction (3.84 Å), adding to the compound's structural fit within the binding pocket. Hydrophobic interactions also play a significant role, with VAL28, ALA75, and ARG21 engaging in alkyl and pi–alkyl interactions (3.83–5.41 Å), which support the van der Waals stabilization (Figure 2). These results prove that *5f* effectively interacts with IsdG, potentially disrupting bacterial iron metabolism and exhibiting antimicrobial activity.

**Figure 3:**
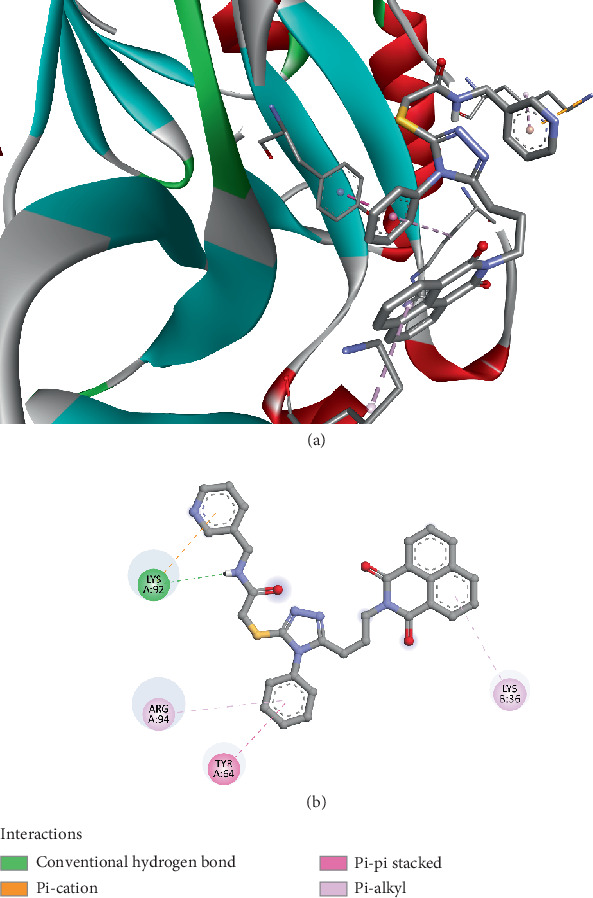
Visualization of molecular docking results showing the interactions between Compound *5f* and the FosB enzyme from *Bacillus cereus* (PDB ID: 8DTD) in both (a) 3D and (b) 2D views.

The molecular docking results of Compound *5f* with the FosB enzyme from *Bacillus cereus* (PDB ID: 8DTD) demonstrated a binding affinity of −6.8 kcal/mol, indicating a moderate yet significant interaction between the ligand and the enzyme's active site. The binding was stabilized by multiple interactions, including a conventional hydrogen bond with LYS92 at a distance of 2.561 Å. This hydrogen bond suggests that LYS92 is a crucial residue for ligand stabilization, as hydrogen bonds often play a vital role in enhancing binding specificity and affinity. An electrostatic pi–cation interaction with LYS92 at 3.947 Å was observed, further reinforcing the ligand's binding within the enzyme pocket. Such electrostatic interactions are essential because they can significantly influence the orientation and stability of the ligand, making it a more effective inhibitor. Hydrophobic interactions also contributed notably to the binding stability. A pi–pi stacked interaction with TYR64 at 3.820 Å indicates aromatic stacking, which is commonly associated with strong, noncovalent interactions that enhance ligand affinity. Furthermore, pi–alkyl interactions were observed with LYS36 (4.938 Å), ARG94 (5.389 Å), and again with LYS92 (4.456 Å) (Figure 3). These hydrophobic contacts suggest that the ligand is well-fitted within the hydrophobic regions of the enzyme's active site, which is critical for maintaining a stable ligand–enzyme complex.

Moreover, the involvement of residues like TYR64 and ARG94, which have been highlighted in prior studies as critical for fosfomycin binding and resistance modulation, further supports the potential efficacy of Compound *5f* [72, 73]. The presence of both hydrogen bonding and hydrophobic interactions in the same binding pocket suggests that Compound *5f* has a dual mechanism of binding stabilization, which could make it more effective than previously tested inhibitors that rely solely on one type of interaction.

The docking results for Compound *5f* indicate a promising potential for inhibiting FosB, offering a pathway to counteracting fosfomycin resistance in *Bacillus cereus*. This compound's stronger binding affinity and multifaceted interaction profile set it apart from earlier inhibitors reported in the literature, suggesting it could be a valuable candidate for further development as an antibiotic adjuvant.

**Figure 4:**
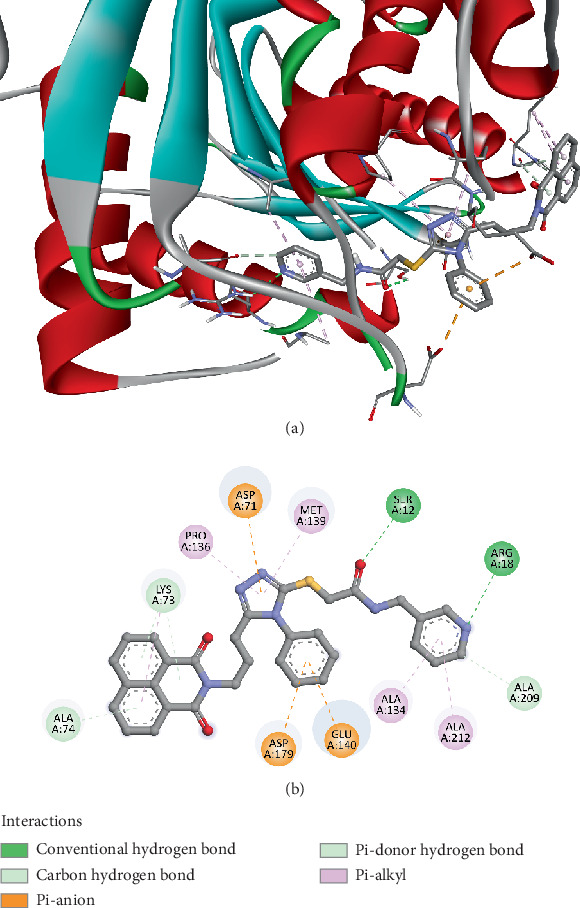
Visualization of molecular docking results showing the interactions between Compound *5f* and the putative cell surface hydrolase from *Lactobacillus plantarum* (PDB ID: 3LP5) in both (a) 3D and (b) 2D views.

Compound *5f* also demonstrated notable binding affinity in molecular docking studies with the putative cell surface hydrolase (3LP5) from *Lactobacillus plantarum*, achieving a docking score of −8.7 kcal/mol. This high binding affinity indicates a strong interaction between the ligand and the protein's active site, suggesting potential biological relevance. The docking analysis revealed multiple conventional hydrogen bonds, including interactions with SER12 and ARG18, at distances of 2.56 and 2.64 Å, respectively, which are well within the optimal hydrogen bonding range, indicating strong, stable interactions. Additionally, ALA209 contributed through a carbon hydrogen bond at 2.85 Å, further stabilizing the ligand within the binding pocket. Electrostatic interactions were also prominent, with GLU140 and ASP179 engaging in pi–anion interactions at distances of 3.88 and 3.84 Å, respectively. These interactions suggest that the negatively charged residues of the hydrolase are effectively interacting with the aromatic rings of Compound *5f*, contributing to binding stability. LYS73 exhibited dual roles, participating in pi–donor hydrogen bonding (2.96 and 3.04 Å) and hydrophobic pi–alkyl interactions (4.33 and 5.28 Å), highlighting the versatility of this residue in stabilizing ligand binding through multiple interaction types. ALA74 also played a dual role with pi–donor hydrogen bonds and pi–alkyl interactions, emphasizing its significance in ligand stabilization. Hydrophobic interactions further reinforced the binding of Compound *5f*, involving residues such as PRO136, MET139, ALA134, and ALA212. These residues formed alkyl and pi–alkyl interactions ranging from 4.27 to 5.31 Å, indicating that the ligand's aromatic and alkyl groups fit snugly into hydrophobic pockets of the enzyme, enhancing the binding affinity (Figure 4). Comprehensive interaction patterns confirm that Compound *5f* effectively engages both polar and nonpolar regions of the binding site, contributing to its strong docking score.

These results are significant when compared to previously published studies on similar triazole-based compounds. Prior research has demonstrated that 1,2,4-triazole derivatives often exhibit potent antimicrobial properties due to their ability to form strong hydrogen bonds and engage in electrostatic interactions with bacterial enzymes. For instance, triazole-based antifungal agents like fluconazole leverage similar binding mechanisms, particularly hydrogen bonding and pi interactions with key residues in fungal enzymes [80]. The strong binding affinity of Compound *5f*, combined with its diverse interaction profile, suggests it could be a promising candidate for further development, potentially exhibiting both antimicrobial efficacy and specificity.

The binding affinity of −8.7 kcal/mol positions Compound *5f* favorably compared to previously investigated ligands targeting the same enzyme, which often exhibit docking scores in the range of −7.2 to −7.6 kcal/mol [81]. This indicates that Compound *5f* could match or exceed the efficacy of existing treatments. Given the enzyme's role in hydrolyzing ester bonds on the bacterial surface, inhibiting 3LP5 could disrupt critical bacterial processes such as adhesion and interaction within the gut microbiome. Since *Lactobacillus plantarum* is a beneficial bacterium, these findings also underscore the importance of assessing potential side effects on the gut microbiota, ensuring that antimicrobial therapies do not inadvertently harm beneficial microbial populations. The docking results for Compound *5f* highlight its strong potential as an antimicrobial agent while emphasizing the need for further experimental validation and safety profiling.

**Figure 5:**
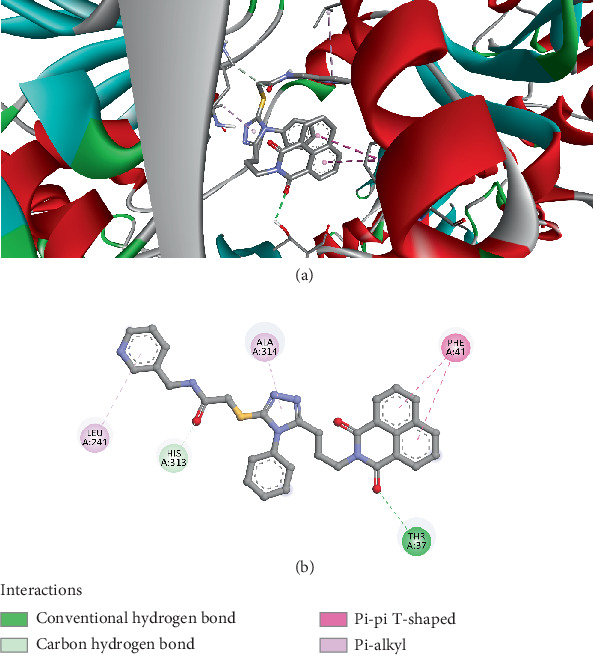
Visualization of molecular docking results showing the interactions between Compound *5f* and the esterase LpEst1 from *Lactobacillus plantarum* (PDB ID: 4C87) in both (a) 3D and (b) 2D views.

The docking results for Compound *5f* with the esterase LpEst1 (PDB ID: 4C87) reveal multiple interactions that contribute to its binding affinity and potential biological activity. The Ligand *5f* binds to the active site of the enzyme with a binding energy of −8.2 kcal/mol, indicating a relatively strong interaction. The results show several key interactions between Compound *5f* and the enzyme, including conventional hydrogen bonds, hydrophobic interactions, and *π*–*π* interactions. The conventional hydrogen bonds are formed between the ligand and amino acids THR37 and HIS313, with bond distances of 2.632 and 3.549 Å, respectively, suggesting a stable interaction within the enzyme's active site. Additionally, there are several hydrophobic interactions: a pi–pi T-shaped interaction with PHE41 at a distance of 5.25 Å and another pi–pi T-shaped interaction with PHE41 at 5.56 Å. These *π*–*π* interactions indicate favorable stacking between the aromatic groups of the ligand and the aromatic residues of the enzyme. Furthermore, an alkyl interaction occurs with ALA314 at 4.03 Å, and a pi–alkyl interaction with LEU241 at 5.28 Å is also observed, contributing to the ligand's overall affinity for the enzyme (Figure 5).

**Figure 6:**
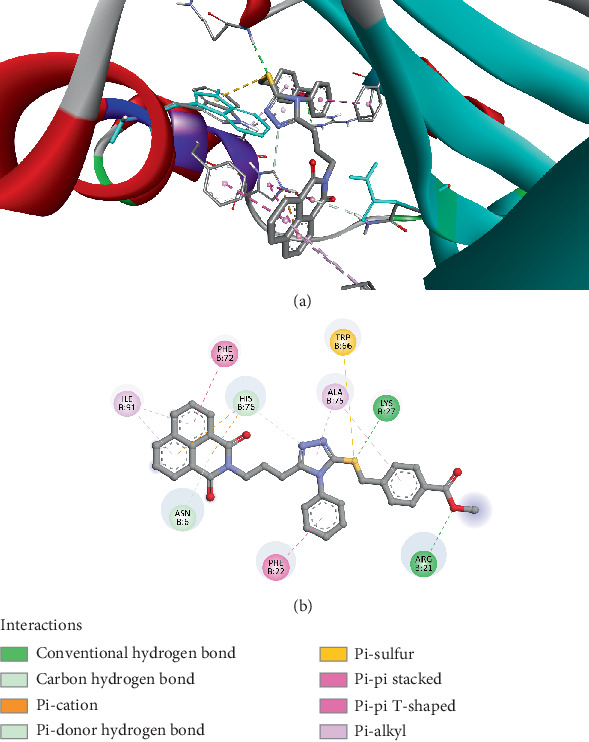
Visualization of molecular docking results showing the interactions between Compound *3e* and the IsdG protein from *Bacillus cereus* (PDB ID: 8AVI) in both (a) 3D and (b) 2D views.

The docking results for Compound *3e* show significant interactions with the IsdG protein target (PDB ID: 8AVI). Several hydrogen bonds are formed between the ligand and key amino acids, including ARG2112, ARG21, LYS27, HIS76, and ASN6, with bond lengths ranging from 2.47 to 2.97 Å. These interactions suggest that the ligand binds effectively to the active site, which is critical for the inhibitory activity of the compound. In addition to hydrogen bonds, the ligand forms electrostatic interactions, including a notable pi–cation interaction with HIS76 (4.01 Å) and a pi–donor hydrogen bond interaction with ASN6 (2.75 Å). These noncovalent interactions play an important role in stabilizing the ligand within the protein's binding site and could contribute to the potency and specificity of the compound's activity. The ligand also interacts with the protein via several hydrophobic interactions, such as pi–pi Stacked with PHE22 (3.81 Å), pi–pi T-shaped with PHE72 (4.75 Å), and pi–alkyl interactions with ALA75, ILE91, and TRP66 (ranging from 3.99 to 5.47 Å) (Figure 6). Hydrophobic interactions are generally critical for stabilizing the ligand in the binding pocket and preventing its dissociation.

The presence of multiple hydrogen bonds and hydrophobic interactions implies a strong affinity between Compound *3e* and IsdG. Additionally, the inclusion of pi–cation and pi–sulfur interactions further supports the compound's potential to interact with aromatic residues, which is a common characteristic for inhibitors targeting bacterial proteins. These docking results suggest that Compound *3e* could be a promising candidate for further development as an IsdG inhibitor. The observed interactions not only demonstrate effective binding but also highlight the versatility of the ligand in forming different types of interactions that contribute to its binding affinity, making it a competitive candidate in the search for potent antibacterial agents targeting IsdG.

**Figure 7:**
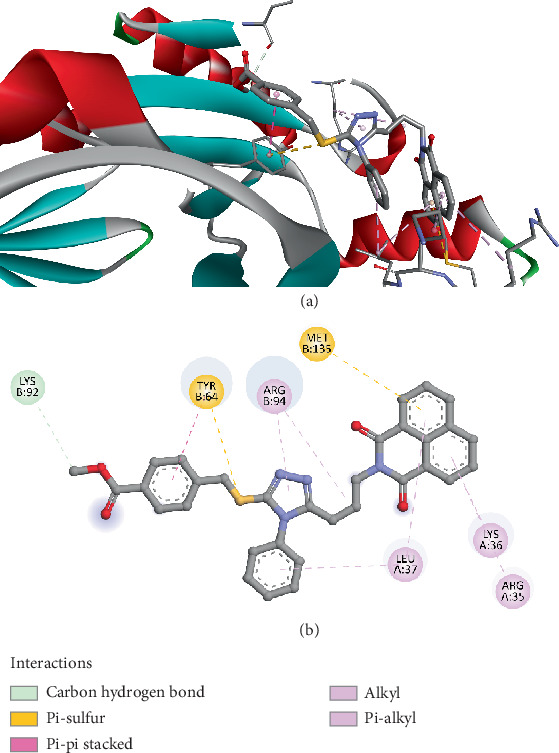
Visualization of molecular docking results showing the interactions between Compound *3e* and the FosB enzyme from *Bacillus cereus* (PDB ID: 8DTD) in both (a) 3D and (b) 2D views.

The docking results for Compound *3e* demonstrate significant interaction with the FosB protein, a transcription factor involved in regulating various cellular processes. The binding affinity of −7.7 kcal/mol suggests a strong interaction between the ligand and the protein. Several important interactions were identified: hydrogen bonding with LYS92, which stabilizes the ligand in the binding pocket; pi–sulfur interactions with MET135 and TYR64, highlighting the importance of aromatic and sulfur-containing residues in the interaction; and hydrophobic interactions, including pi–pi stacking between the ligand's aromatic rings and the protein's aromatic residues. The pi–alkyl interactions with ARG35, LYS36, LEU37, and ARG94 further reinforce the stability of the ligand–protein complex (Figure 7). These interactions suggest that Compound *3e* binds effectively to the protein's active site, possibly inhibiting its function by blocking essential binding pockets or active sites.

**Figure 8:**
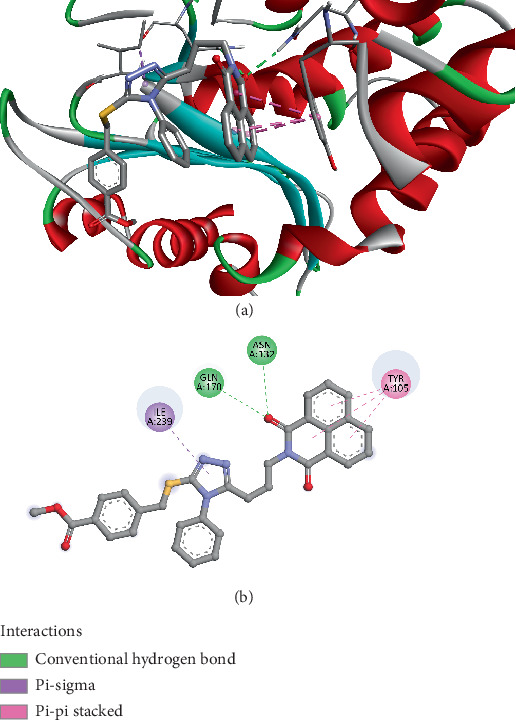
Visualization of molecular docking results showing the interactions between Compound *3e* and the dihydrofolate reductase from *Staphylococcus aureus* (PDB ID: 1DHI) in both (a) 3D and (b) 2D views.

The docking results of Compound *3e* with DHFR reveal several notable interactions that may indicate a strong binding affinity between the ligand and the enzyme. The binding affinity score for Compound *3e* is −7.3 kcal/mol, which suggests a moderate to strong interaction with the target. The interactions include several hydrogen bonds and hydrophobic interactions, contributing to the stability of the complex. Specifically, Compound *3e* forms conventional hydrogen bonds with ASN132 (2.33 Å) and GLN170 (2.60 Å), which are both critical residues in the binding pocket of DHFR. These hydrogen bonds suggest that Compound *3e* can establish strong polar interactions with key amino acids in the enzyme's active site, facilitating the ligand's binding and potentially influencing the enzyme's functionality. Additionally, Compound 3e interacts with TYR105 through hydrophobic pi–pi stacking interactions (3.68, 4.15, and 4.06 Å), which further stabilize the binding. The presence of these stacking interactions may enhance the specificity and affinity of the ligand for DHFR, as aromatic residues like TYR105 are known to play a significant role in stabilizing ligand binding in the active site through aromatic–aromatic interactions. Another hydrophobic interaction occurs with ILE239 via an alkyl interaction (4.82 Å), further stabilizing the complex (Figure 8). The hydrophobic interactions are crucial for the ligand's fit within the hydrophobic core of the enzyme's binding pocket, and they often contribute to the overall binding strength by reducing the desolvation energy.

These docking results suggest that Compound *3e* has a promising binding profile for DHFR, supported by both polar and hydrophobic interactions. The presence of multiple hydrogen bonds and pi–pi stacking interactions is a positive indicator of its potential efficacy as an inhibitor of DHFR [82]. Compared to previously published research on DHFR inhibitors, such as the studies on antifolate drugs [83] (e.g., methotrexate) and triazole derivatives [84], these results align well with the established binding modes. Methotrexate, for instance, also forms hydrogen bonds with similar residues like ASN132 and GLN170 in DHFR and utilizes hydrophobic interactions to stabilize the binding. The observed interactions in this study with Compound *3e* could be comparable in terms of the strength and type of interactions, but the presence of the unique 1,2,4-triazole ring with specific substituents at the fifth, third, and fourth positions introduces novel features that may offer unique advantages in terms of selectivity and potency. Further experimental validation would be required to confirm the inhibitor's potential and its efficacy in biological systems.

**Figure 9:**
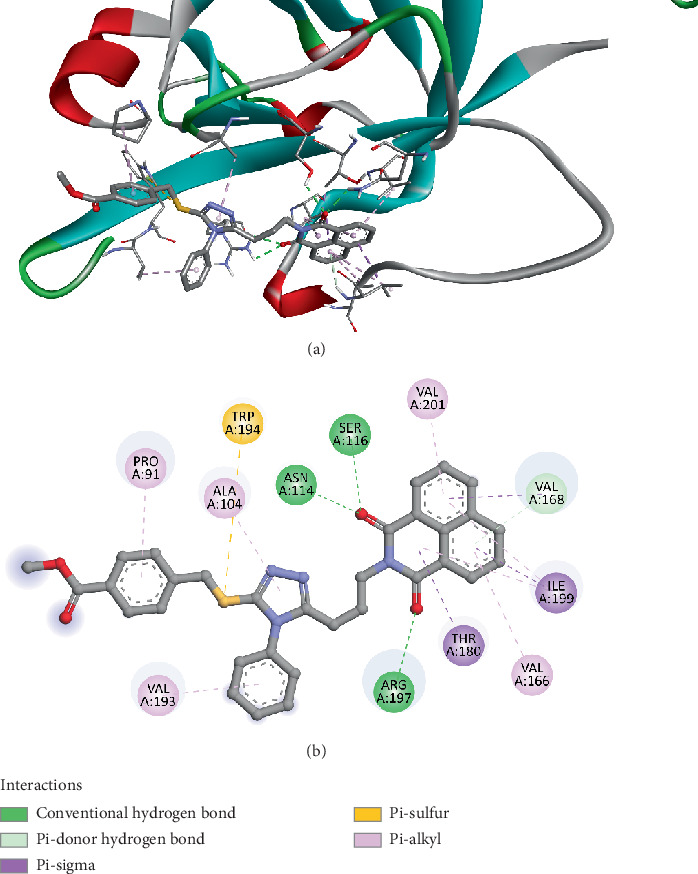
Visualization of molecular docking results showing the interactions between Compound *3e* and the enzyme Sortase A from *Staphylococcus aureus* (PDB ID: 1T2P) in both (a) 3D and (b) 2D views.

The molecular docking analysis of Compound *3e* targeting Sortase A (PDB ID: 1T2P) revealed a strong binding affinity of −9.8 kcal/mol, suggesting a high potential for interaction and inhibitory activity. Hydrogen bonding interactions play a significant role in anchoring Compound *3e*, with key residues ASN114, SER116, and ARG197 forming conventional hydrogen bonds at distances of 2.19, 2.44, 2.48, and 2.43 Å, respectively. Additional hydrogen bonding is observed with VAL168 (2.95 Å), which further stabilizes the ligand–receptor complex. Beyond hydrogen bonding, *π*-based interactions contribute to the strong binding affinity of Compound *3e*. Pi–donor hydrogen bond formation with VAL168 (3.93 Å) and pi–sigma interactions with THR180 (3.81 Å), ILE199 (3.82 Å), and TRP194 (5.10 Å) indicate that Compound *3e* engages in aromatic interactions commonly found in effective drug-receptor binding. These interactions enhance the binding stability and specificity, supporting the potential for Sortase A inhibition. Notably, TRP194 also forms a pi–sulfur interaction (5.11 Å), which may further reinforce the binding through noncovalent sulfur-based stabilization. The docking results also reveal multiple hydrophobic interactions, which are crucial for van der Waals stabilization and increasing ligand retention in the active site. Residues such as ALA104, ILE199, VAL201, VAL168, VAL193, PRO91, and AL166 participate in pi–alkyl interactions ranging from 4.24 to 5.49 Å, indicating that Compound *3e* establishes substantial nonpolar contacts (Figure 9). These interactions further strengthen ligand stability, reducing the likelihood of dissociation from the enzyme's active site. Results showed that Compound *3e* is well suited for binding to Sortase A and may act as an effective inhibitor.

In comparison to previous studies, the docking results of Compound *3e* show promising similarities to other reported inhibitors of Sortase A, which also form multiple hydrogen bonds and hydrophobic interactions with the enzyme's active site residues. For instance, similar interactions have been observed in studies of Sortase A inhibitors, where inhibitors form hydrogen bonds with residues such as ASN114 and ARG197 [85–87], and hydrophobic interactions with residues like TRP194 and ILE199 [88–90]. These interactions have been linked to effective inhibition of Sortase A, making Compound *3e* a promising candidate for further experimental validation. Moreover, the docking score of −9.8 kcal/mol suggests a strong binding affinity, which is consistent with the results observed in other Sortase A inhibitors. This score indicates that Compound *3e* could potentially act as a potent inhibitor of Sortase A, supporting its potential application in drug development targeting bacterial infections where Sortase A plays a crucial role in the virulence of pathogens. In conclusion, the docking results for Compound *3e* reveal that it binds efficiently to Sortase A through multiple strong interactions, including hydrogen bonds, hydrophobic interactions, and pi interactions. These findings align with previous research on Sortase A inhibitors, suggesting that Compound *3e* could be a valuable lead compound for the development of new antimicrobial agents. Further in vitro and in vivo studies will be necessary to confirm the efficacy of this compound as a Sortase A inhibitor.

**Figure 10:**
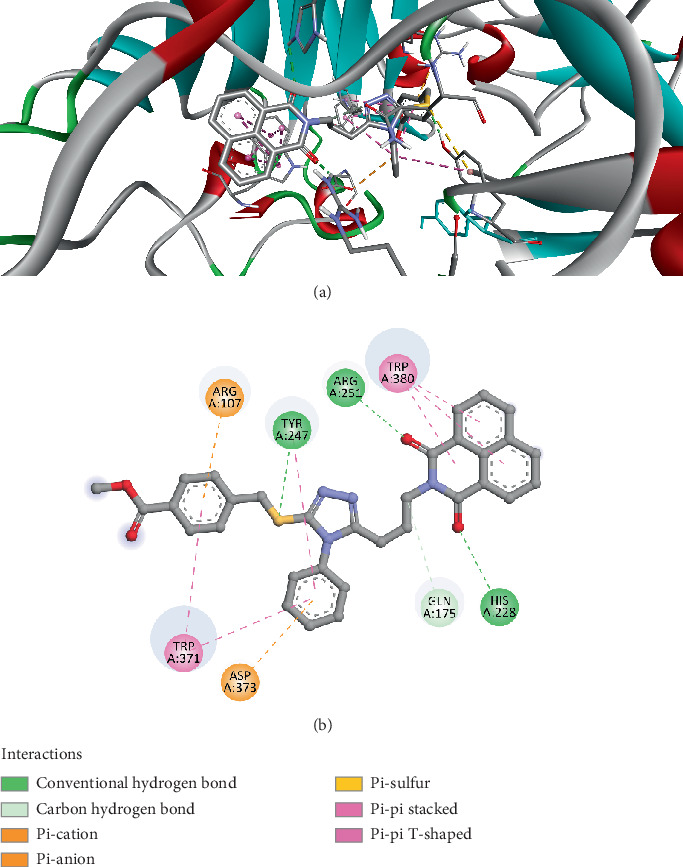
Visualization of molecular docking results showing the interactions between Compound *3e* and Cel7A from *Geotrichum candidum* (PDB ID: 4ZZT) in both (a) 3D and (b) 2D views.

The docking results for Compound *3e* against the Cel7A structure (PDB ID: 4ZZT) provide a detailed analysis of the interactions between the ligand and the protein. The ligand forms several significant interactions with the binding site residues, primarily hydrogen bonds, electrostatic interactions, and hydrophobic contacts (Figure 10). The hydrogen bonds involve key residues such as HIS228, TYR247, ARG251, GLN175, and ARG107, with distances ranging from 2.15 to 3.21 Å. These hydrogen bonds, both conventional and carbon–hydrogen bonds, indicate strong interactions that could contribute to the stability and binding affinity of the ligand to the target protein. Furthermore, electrostatic interactions, including pi–cation and pi–anion interactions, are observed with residues like ASP373 and TYR247. The pi–cation interaction at a distance of 3.73 Å with ARG107 and the pi–anion interaction with ASP373 at 4.17 Å could suggest specific stabilization of the ligand within the active site. Additionally, a pi–sulfur interaction with TYR247 at 5.14 Å further supports the possibility of aromatic and sulfur-related interactions, which are crucial in stabilizing the ligand–protein complex. The hydrophobic interactions observed in the docking results are extensive, with multiple pi–pi stacking interactions occurring with residues such as TRP371 and TRP380 at distances ranging from 4.22 to 5.58 Å. These interactions highlight the importance of aromatic stacking in the ligand's binding mode. The pi–pi T-shaped interaction with TRP371 at 5.57 and 5.47 Å indicates that the ligand's aromatic rings align well with the aromatic residues in the protein's active site. Hydrophobic alkyl interactions with PRO177 at 4.74 Å further contribute to the overall binding strength. These results indicate that Compound *3e* forms a stable and strong binding complex with the Cel7A enzyme, primarily driven by hydrogen bonding, electrostatic, and hydrophobic interactions. The extensive pi–pi stacking interactions observed in this docking study are particularly noteworthy, as they suggest a significant contribution of aromatic interactions to the ligand's binding.

Comparing these results with previously published research, several studies have shown similar types of interactions between lSigands and the Cel7A enzyme. For instance, previous docking studies have also highlighted the importance of hydrogen bonds and hydrophobic interactions in stabilizing ligand–protein complexes [91]. The presence of pi–cation and pi–anion interactions is in line with other studies that have investigated the role of aromatic interactions in enhancing the binding affinity of small molecules to glycoside hydrolase enzymes. The docking results for Compound *3e* against Cel7A suggest that it binds strongly to the enzyme, with multiple interaction types contributing to its overall affinity. These interactions, particularly hydrogen bonds, pi-stacking, and electrostatic contacts, are consistent with findings in the literature, confirming the compound's potential as a promising inhibitor of Cel7A.

**Figure 11:**
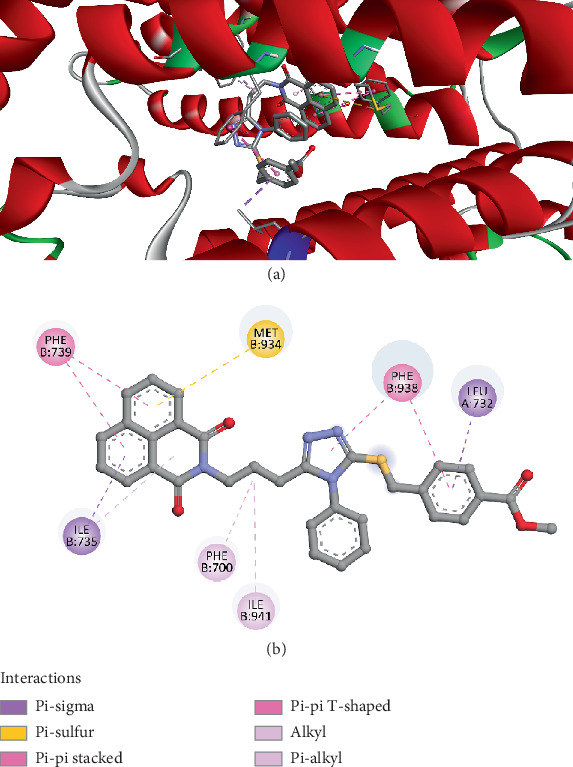
Visualization of molecular docking results showing the interactions between Compound *3e* and the Chitin Synthase 2 from *Geotrichum candidum* (PDB ID: 7STO) in both (a) 3D and (b) 2D views.

The molecular docking analysis of Compound *3e* with Chitin Synthase 2 (PDB ID: 7STO) revealed a binding affinity of −9.1 kcal/mol, indicating a strong interaction between the ligand and the enzyme's active site. The docking results highlight *π* interactions, sulfur-based stabilization, and hydrophobic forces, which collectively enhance the binding stability and may contribute to the potential inhibitory activity of Compound *3e* (Figure 11). Key *π* interactions play a crucial role in the ligand's binding stability. Pi–sigma interactions were observed with ILE735 (3.62 Å) and LEU732 (3.63 Å), which help position the ligand within the enzyme's active site, facilitating stronger binding. Additionally, pi–pi stacking interactions with PHE739 (4.92 and 5.30 Å) and pi–pi T-shaped interactions with PHE938 (4.75 and 5.29 Å) suggest that the compound establishes favorable aromatic interactions, commonly associated with strong and selective binding in drug-target interactions. A significant pi–sulfur interaction was detected with MET934 (4.96 Å), which may contribute to enhanced binding stability by leveraging the sulfur atom's polarizability, potentially increasing specificity toward Chitin Synthase 2. This type of interaction is often found in enzyme–ligand complexes where sulfur-containing residues contribute to structural stabilization. Hydrophobic interactions were observed, reinforcing the hydrophobic pocket around the ligand. Alkyl interactions with ILE941 (4.60 Å), as well as pi–alkyl interactions with PHE700 (3.87 Å) and ILE735 (4.85 Å), indicate additional van der Waals forces that strengthen the ligand's retention within the enzyme's active site. The docking results suggest that Compound *3e* forms a stable and well-anchored complex with Chitin Synthase 2 through a combination of *π*-stacking, sulfur-based interactions, and hydrophobic forces. These favorable binding characteristics imply that Compound *3e* has the potential to act as an effective inhibitor of Chitin Synthase 2, warranting further in vitro and in vivo studies to explore its potential applications in fungal or insect chitin biosynthesis inhibition.

In conclusion, the molecular docking results highlight the strong binding affinities and diverse interaction profiles of Compound *5f* across multiple bacterial enzymes, including IsdG and FosB from *Bacillus cereus*, putative cell surface hydrolase (3LP5) from *Lactobacillus plantarum*, and esterase LpEst1. The combination of hydrogen bonding, electrostatic, and hydrophobic interactions suggests that Compound *5f* has significant potential as an antibacterial agent, particularly in targeting iron-regulated pathways and fosfomycin resistance mechanisms. Additionally, Compound *3e* demonstrated notable binding characteristics, further supporting the relevance of 1,2,4-triazole derivatives in antimicrobial research. While molecular docking offers valuable predictive insights, it does not confirm biological activity. Therefore, in vitro and in vivo validation of binding interactions is essential in future studies.

## 4. Conclusion

The synthesis and evaluation of novel 1,2,4-triazole derivatives demonstrated a strategic and efficient approach to obtaining structurally diverse compounds with potential biological applications via classical and green approaches. Biological screening revealed notable antimicrobial activity among selected compounds. Specifically, Compound *5f* exhibited strong broad-spectrum activity, particularly against *Bacillus cereus* and *Lactobacillus plantarum*, suggesting its potential as a promising antimicrobial agent. Similarly, Compound *3e* demonstrated significant efficacy against multiple microbial strains, including *Geotrichum candidum* and *Staphylococcus aureus*, highlighting its dual-action antimicrobial and antifungal potential. The study also identified key structural features influencing antimicrobial potency. The presence of pyridinyl and benzoate moieties in Compounds *5f* and *3e*, respectively, appeared to enhance their activity, likely through improved binding interactions with microbial targets and enhanced lipophilicity. These findings provide valuable insights into the SARs of 1,2,4-triazole derivatives, guiding future efforts in rational drug design. The synthesized compounds were assessed for their anti-inflammatory activity through IL-6 inhibition assays. The results suggested that select compounds exhibited potential in modulating inflammatory responses, warranting further investigation into their therapeutic applications.

Overall, this study highlights the promising biological potential of 1,2,4-triazole derivatives, particularly in antimicrobial and anti-inflammatory applications. Future work should focus on elucidating their mechanisms of action, optimizing their pharmacokinetic properties, and conducting in vivo evaluations to explore their clinical viability. These findings contribute to the growing body of research on 1,2,4-triazoles as versatile bioactive scaffolds with potential therapeutic significance.

## Figures and Tables

**Table 1:** Antagonistic activity of organic compounds against test strains of microorganisms.

**Concentration (CFU/mL)**																	
	**Tested microorganisms**	**Starting concentration м/о**	**Control**	**1**	**3a**	**3b**	**3c**	**3d**	**3e**	**4**	**5a**	**5b**	**5c**	**5d**	**5e**	**5f**	**5g**
1	*Enterococcus faecalis*	1.5 · 10^8^	ND	ND	ND	ND	ND	ND	ND	ND	ND	ND	ND	ND	ND	ND	ND
2	*Escherichia coli (lac+)*	1.5 · 10^8^	ND	ND	ND	ND	ND	ND	ND	ND	ND	ND	ND	ND	ND	ND	ND
3	*Klebsiella oxytoca*	1.5 · 10^8^	ND	ND	ND	ND	ND	ND	ND	ND	ND	ND	ND	ND	ND	ND	ND
4	*Pseudomonas aeruginosa*	1.5 · 10^8^	ND	ND	ND	ND	ND	ND	ND	ND	ND	ND	ND	ND	ND	ND	ND
5	*Staphylococcus aureus*	1.5 · 10^8^	1 · 10^6^	ND	ND	ND	ND	ND	1·10^6^	ND	ND	ND	ND	ND	ND	ND	ND
6	*Bacillus cereus ATCC 11778*	1.5 · 10^8^	5 · 10^8^	4 · 10^8^	2 · 10^8^	2 · 10^8^	2 · 10^8^	1 · 10^8^	1 · 10^8^	3 · 10^7^	2 · 10^8^	5 · 10^8^	3 · 10^8^	2 · 10^8^	1 · 10^10^	10^6^	2 · 10^8^
7	*Geotrihium candidum*	1.5 · 10^8^	2.4 · 10^7^	ND	ND	ND	ND	ND	2.4 · 10^7^	ND	ND	ND	ND	ND	ND	ND	ND
8	*Candida albicans*	1.5 · 10^8^	ND	ND	ND	ND	ND	ND	ND	ND	ND	ND	ND	ND	ND	ND	ND
9	*Lactobacillus plantarum A*	1.5 · 10^8^	ND	ND	ND	ND	ND	ND	ND	ND	1 · 10^10^	ND	ND	ND	ND	1 · 10^8^	1 · 10^4^
10	*Serratia ficaria*	1.5 · 10^8^	ND	ND	ND	ND	ND	ND	ND	ND	ND	ND	ND	ND	ND	ND	ND

**Table 2:** Anti-inflammatory activity of synthesized compounds as determined by IL-6 levels (picograms per milliliter).

**Compound**	**IL-6 value (pg/mL)**	**Control (material without compound) (pg/mL)**	**Presence or absence of activity**
1	12	12	Not active
3a	7	12	Active
3b	12	12	Not active
3c	8	12	Active
3d	10	12	Active
3e	8	12	Active
4	8	12	Active
5a	10	12	Active
5b	11	12	Not active
5c	7	12	Active
5d	9	12	Active
5e	9	12	Active
5f	8	12	Active
5g	8	12	Active

**Table 3:** Molecular docking interactions and binding affinities for selected compounds with target proteins.

**Protein ID**	**Protein Name**	**Ligand**	**Affinity (kcal/mol)**	**Interacting amino acids (residue)**	**Bond type**
8AVI	IsdG	5f	−10.2	GLN79	Conventional hydrogen bond
				HIS76	Pi–cation and pi–donor H bond
				ILE53	Pi–sigma
				HIS76	Pi–Pi stacked
				PHE22	Pi–Pi T-shaped (four contacts)
				VAL28 and ALA75	Alkyl
				ARG21 and ALA75	Pi–alkyl

8AVI	IsdG	3e	−9.8	ARG2112, ARG21, and LYS27	Conventional hydrogen bond
				HIS76	Electrostatic, pi–cation, and pi–donor H bond
				ASN6	Pi–donor hydrogen bond
				TRP66, PHE22, PHE72, and HIS76	Hydrophobic (pi–pi stacked and T-shaped)
				ALA75, TRP66, and ILE91	Hydrophobic pi–alkyl

8DTD	FosB	5f	−6.8	LYS92	Conventional H bond and electrostatic pi–cation
				TYR64, LYS36, and ARG94	Hydrophobic pi–pi stacked/pi–alkyl

8DTD	FosB	3e	−7.7	LYS92, MET135, and TYR64	Carbon H bond and pi–sulfur
				TYR64, ARG94, LEU37, and ARG35	Hydrophobic pi–pi stacked/alkyl/pi–alkyl

3LP5	Putative cell surface hydrolase	5f	−8.7	SER12 and ARG18	Conventional hydrogen bond
				ALA209	Carbon hydrogen bond
				GLU140 and ASP179	Electrostatic pi–anion
				LYS73 and ALA74	Pi–donor hydrogen bond
				PRO136, MET139, LYS73, ALA74, ALA134, and ALA212	Hydrophobic alkyl/pi–alkyl

4C87	Esterase LpEst1	5f	−8.2	THR37 and HIS313	Conventional and carbon H bond
				PHE41	Hydrophobic pi–pi T-shaped
				ALA314 and LEU241	Hydrophobic alkyl/pi–alkyl

1DHI	Dihydrofolate reductase	3e	−7.3	ASN132 and GLN170	Conventional hydrogen bond
				TYR105	Hydrophobic pi–pi stacked (three contacts)
				ILE239	Hydrophobic alkyl

1T2P	Sortase A	3e	−9.8	ASN114, SER116, and ARG197	Conventional hydrogen bond
				VAL168	Pi–donor H bond, pi–sigma, and pi–alkyl
				THR180 and ILE199	Pi–sigma
				TRP194	Pi–sulfur
				ALA104, ILE199, VAL201, VAL168, VAL193, and PRO91	Pi–alkyl

4ZZT	Cel7A structure	3e	−13.1	HIS228, TYR247, and ARG251	Conventional hydrogen bond
				GLN175	Carbon hydrogen bond
				ARG107	Electrostatic pi–cation
				ASP373	Electrostatic pi–anion
				TYR247, TRP371, and TRP380	Hydrophobic pi–pi stacked
				PRO177	Hydrophobic alkyl

7STO	Chitin Synthase 2	3e	−9.1	ILE735 and LEU732	Pi–sigma
				MET934	Pi–sulfur
				PHE739 and PHE938	Pi–pi stacked/T-shaped
				ILE941	Alkyl
				PHE700 and ILE735	Pi–alkyl

## Data Availability

The data that support the findings of this study are available from the corresponding author upon reasonable request.
