# Supplementary figures and images for: Design and Enzyme-Targeted Assessment of 1,2,4-Triazole–1,8-Naphthalimide Hybrids in Drug Discovery
Source: Biomed Res Int. 2025 Aug 10;2025:6115993. doi: 10.1155/bmri/6115993 (PMC12358230; doi:10.1155/bmri/6115993)

BE52425260

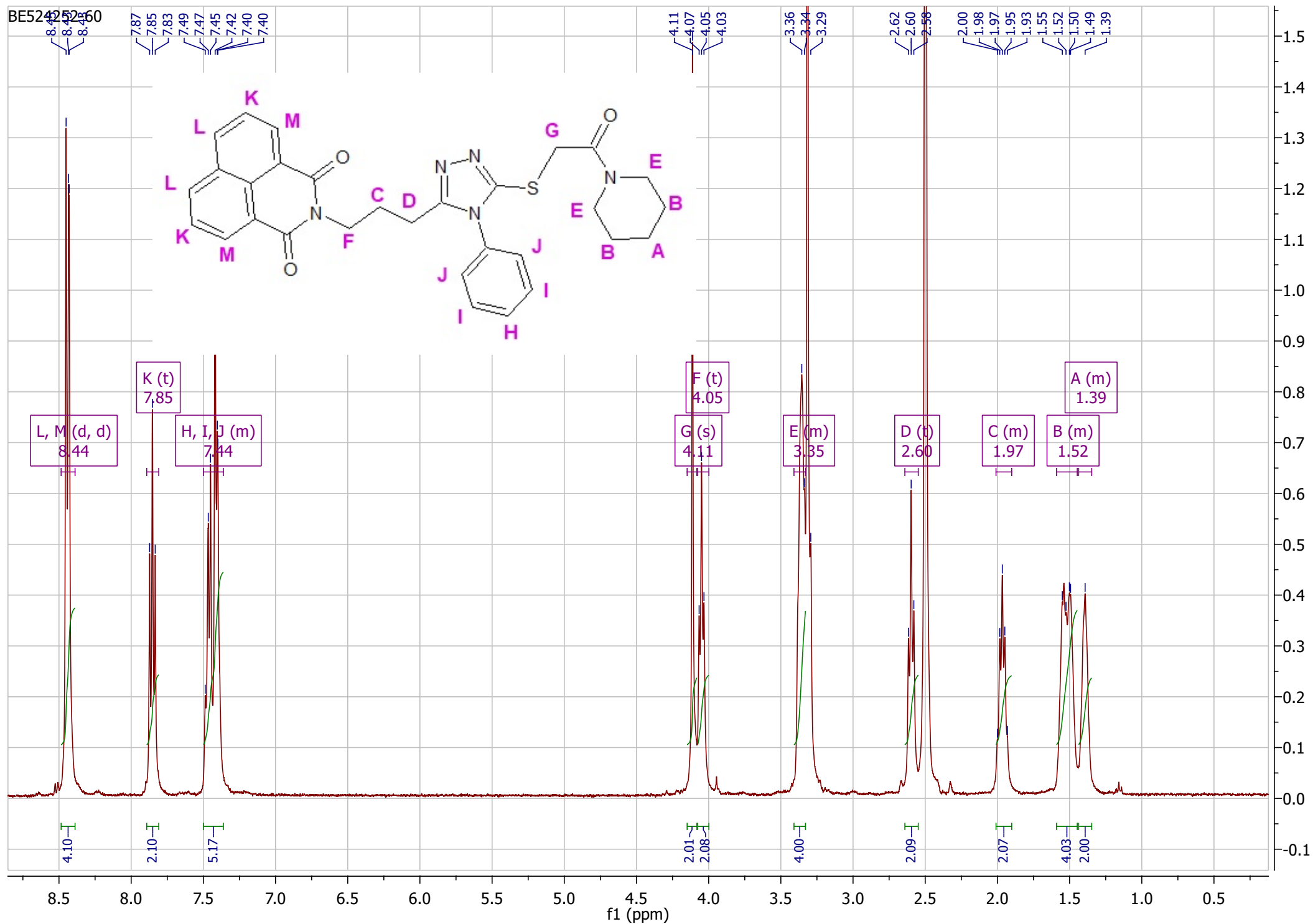

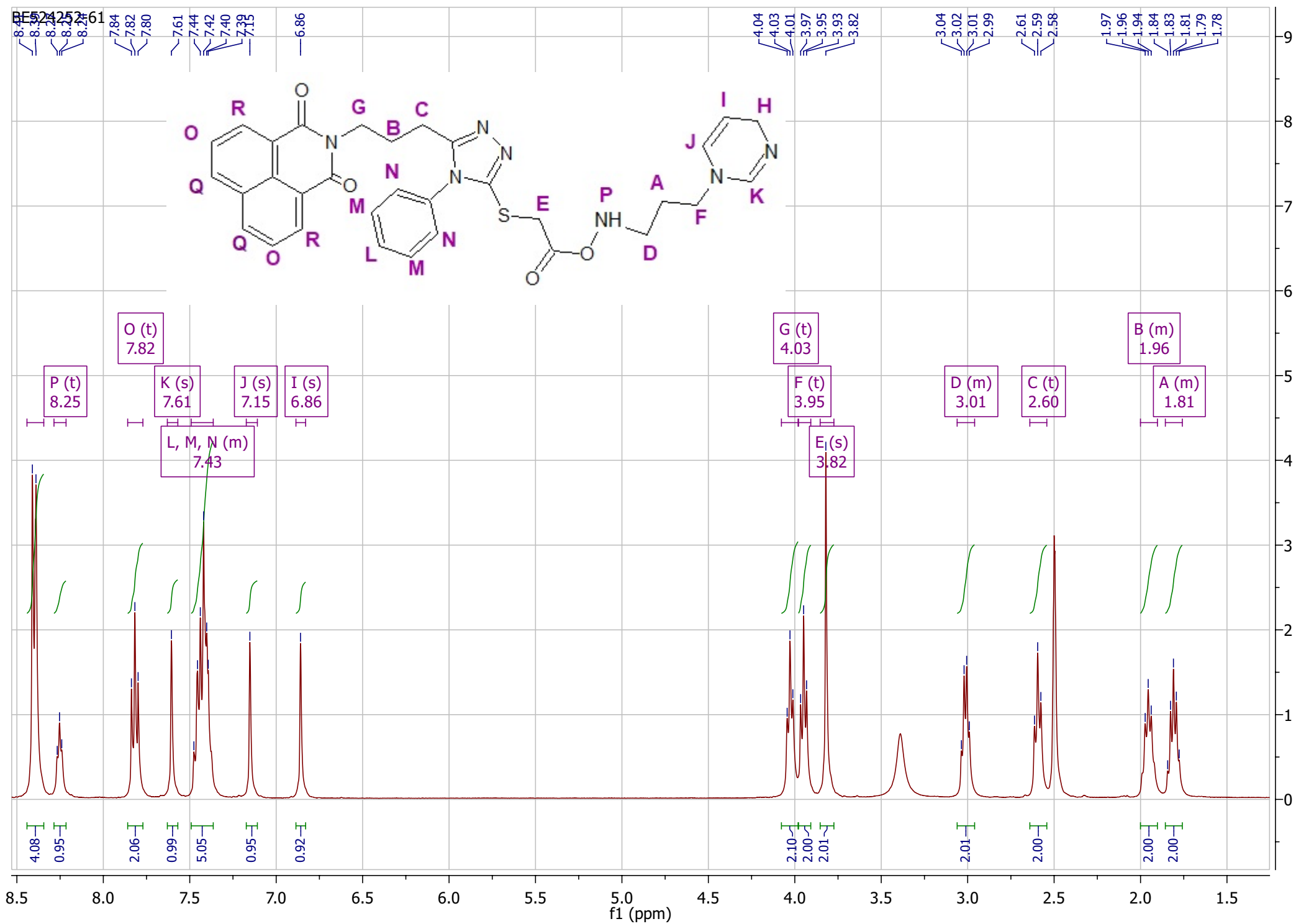

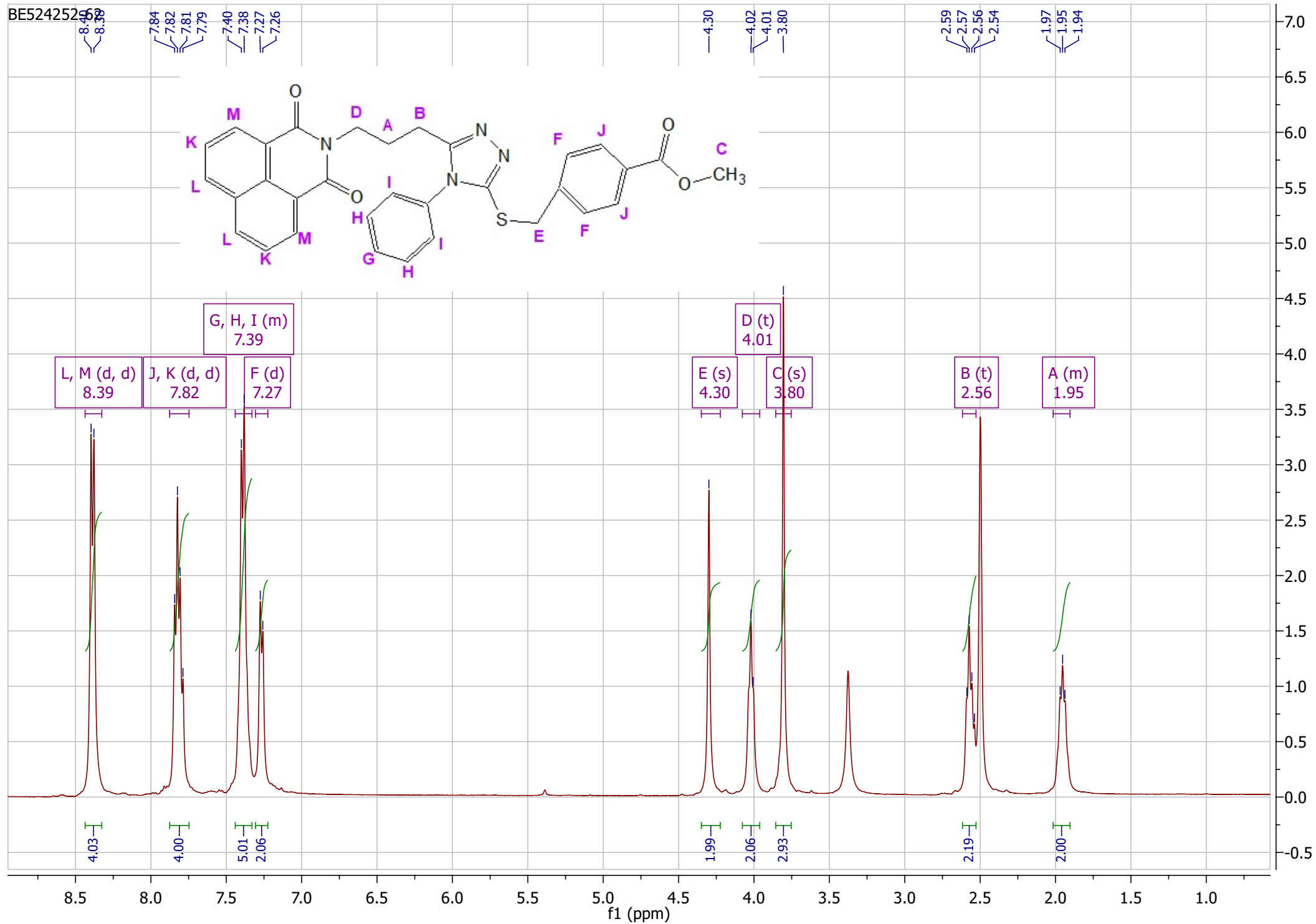

BE524252-63

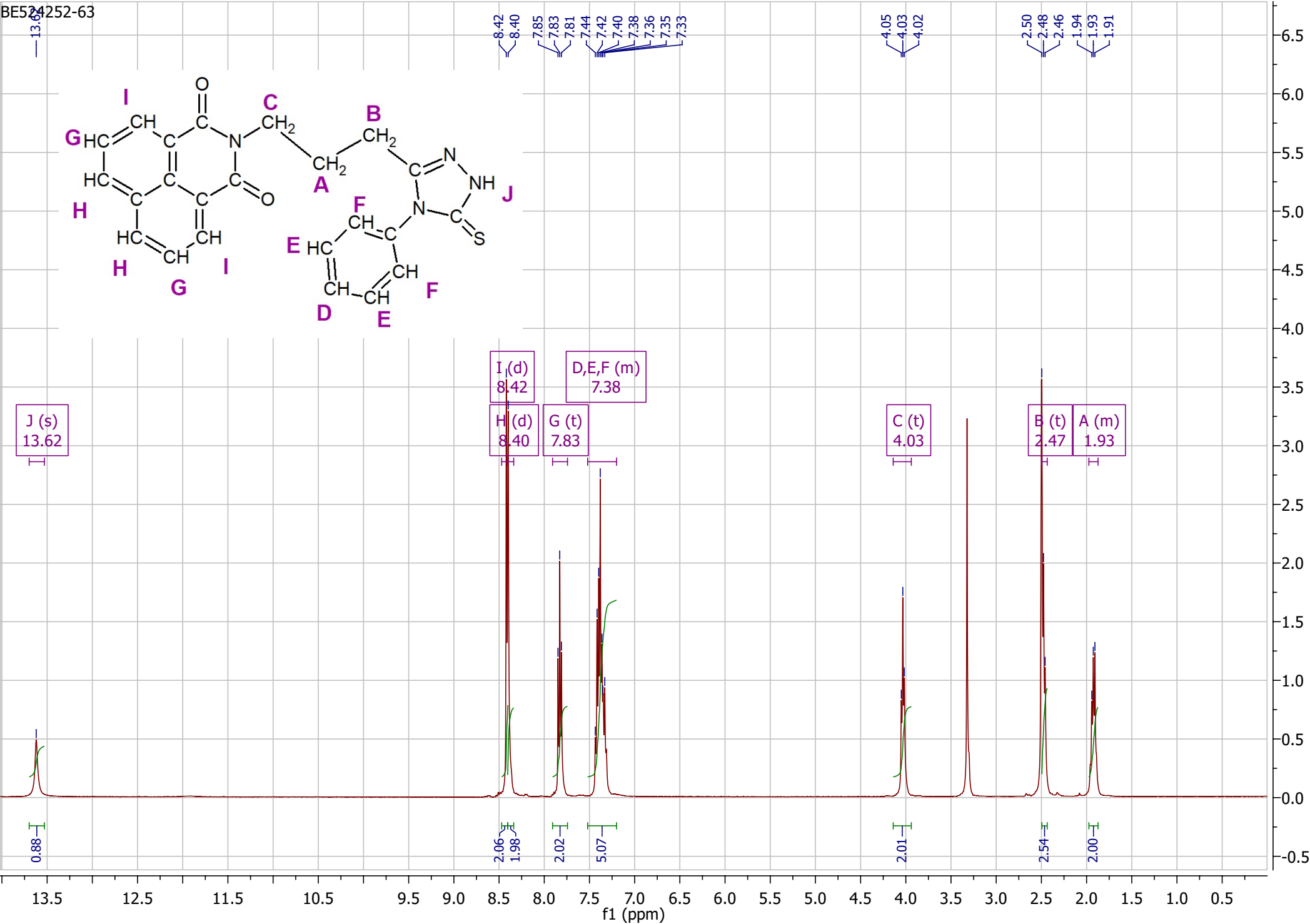

BE524252-64

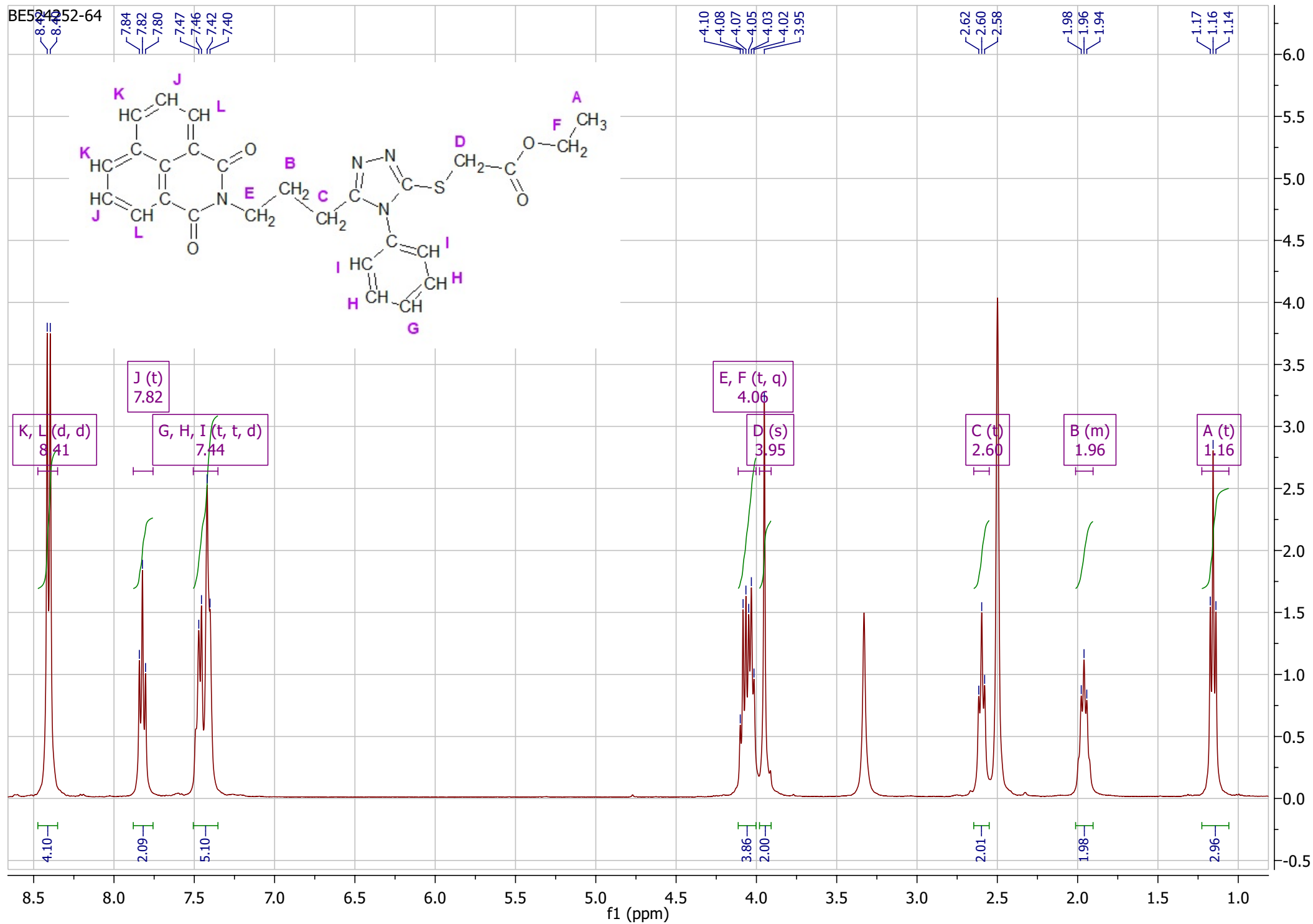

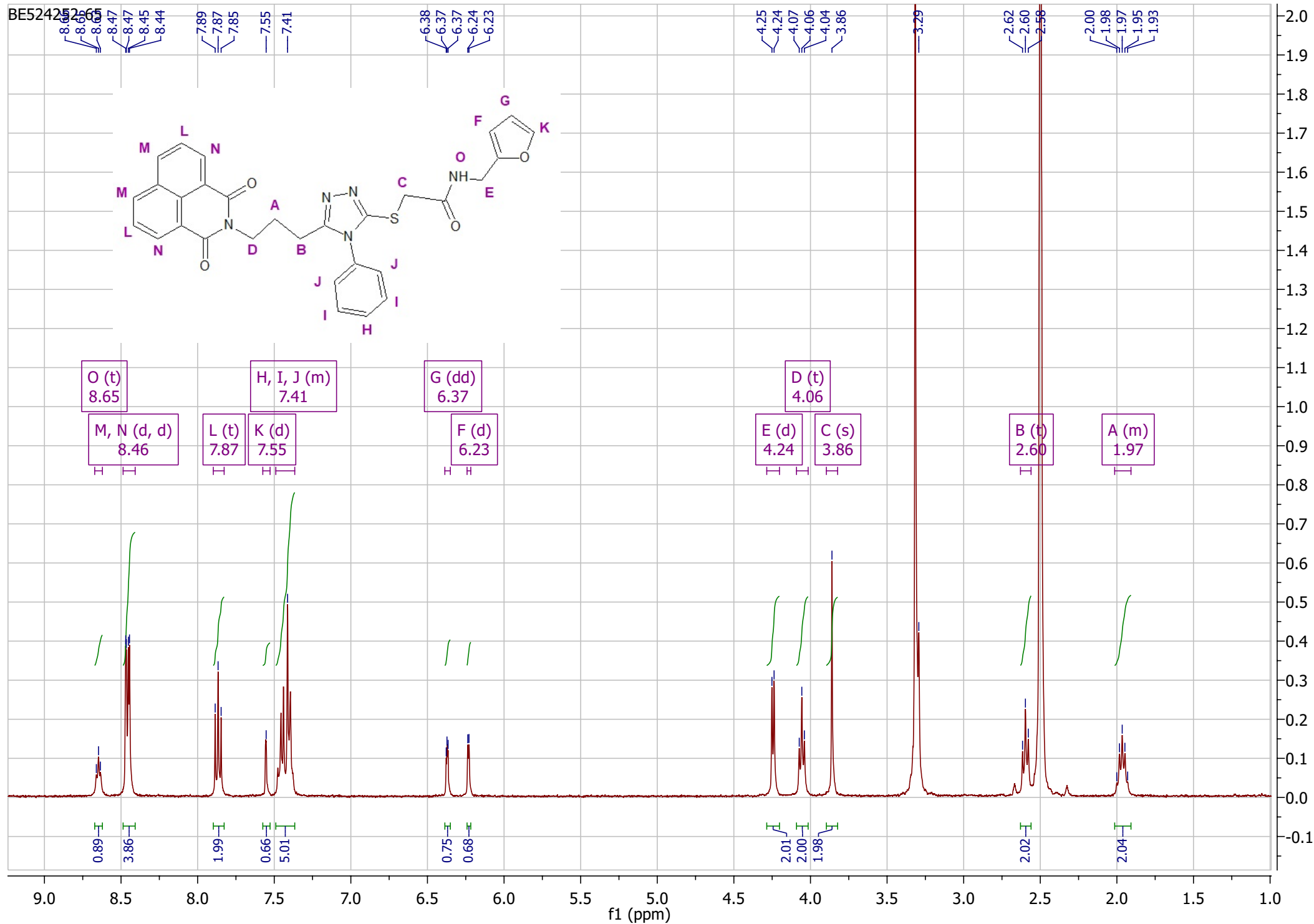

BE524252-66

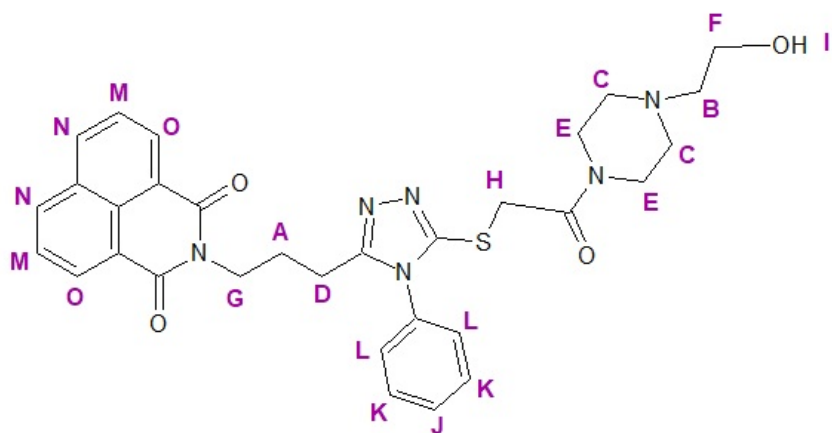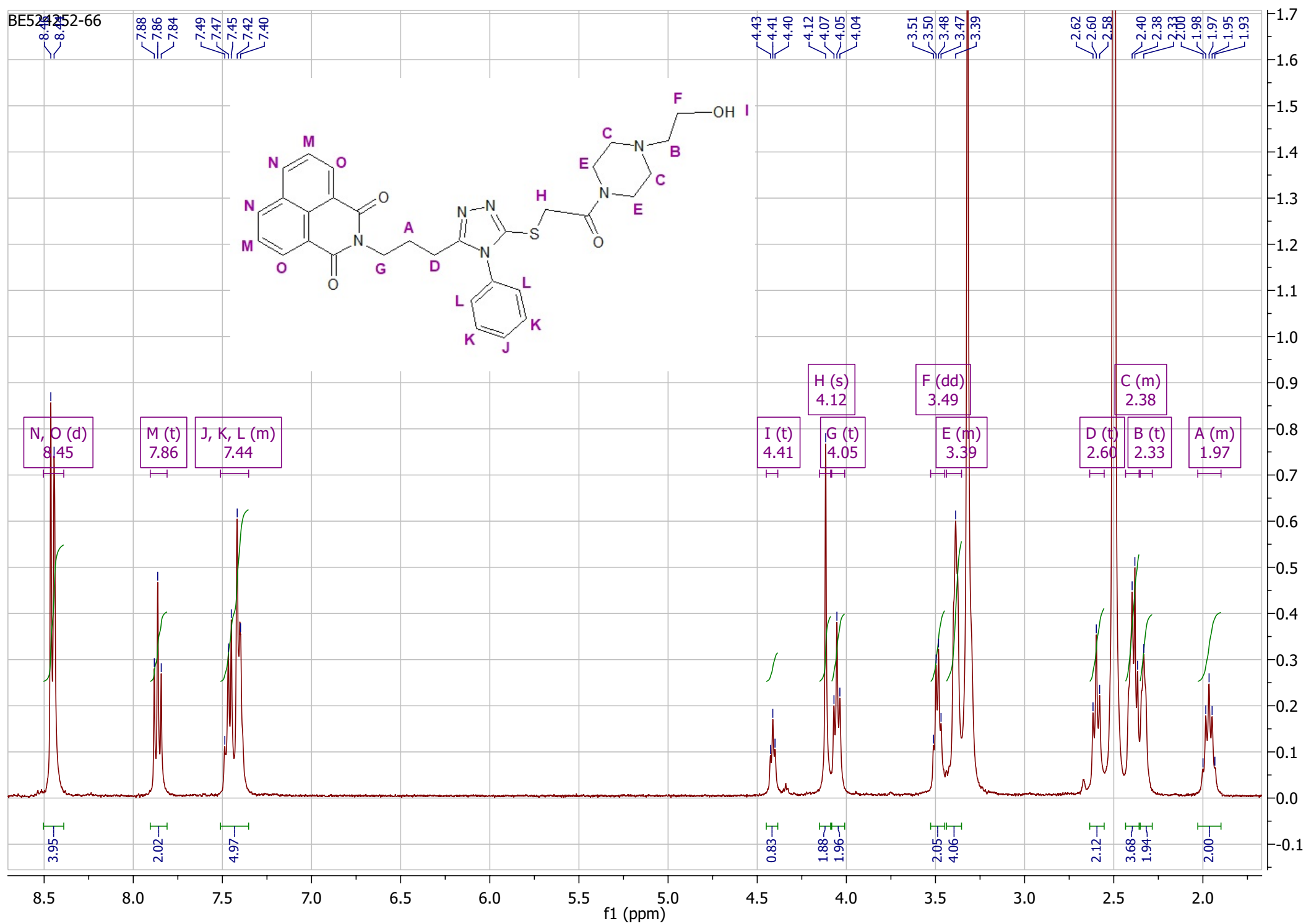

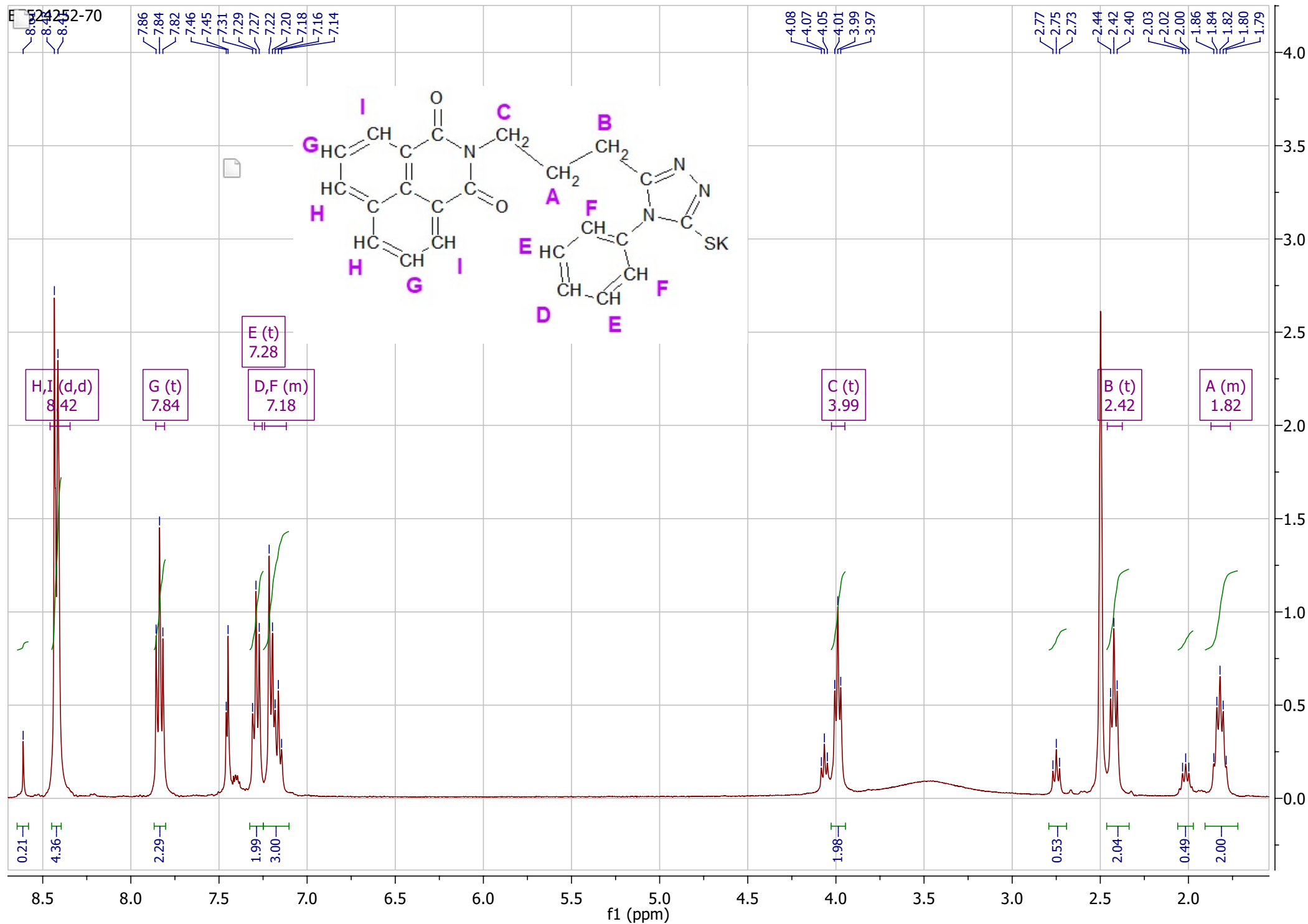

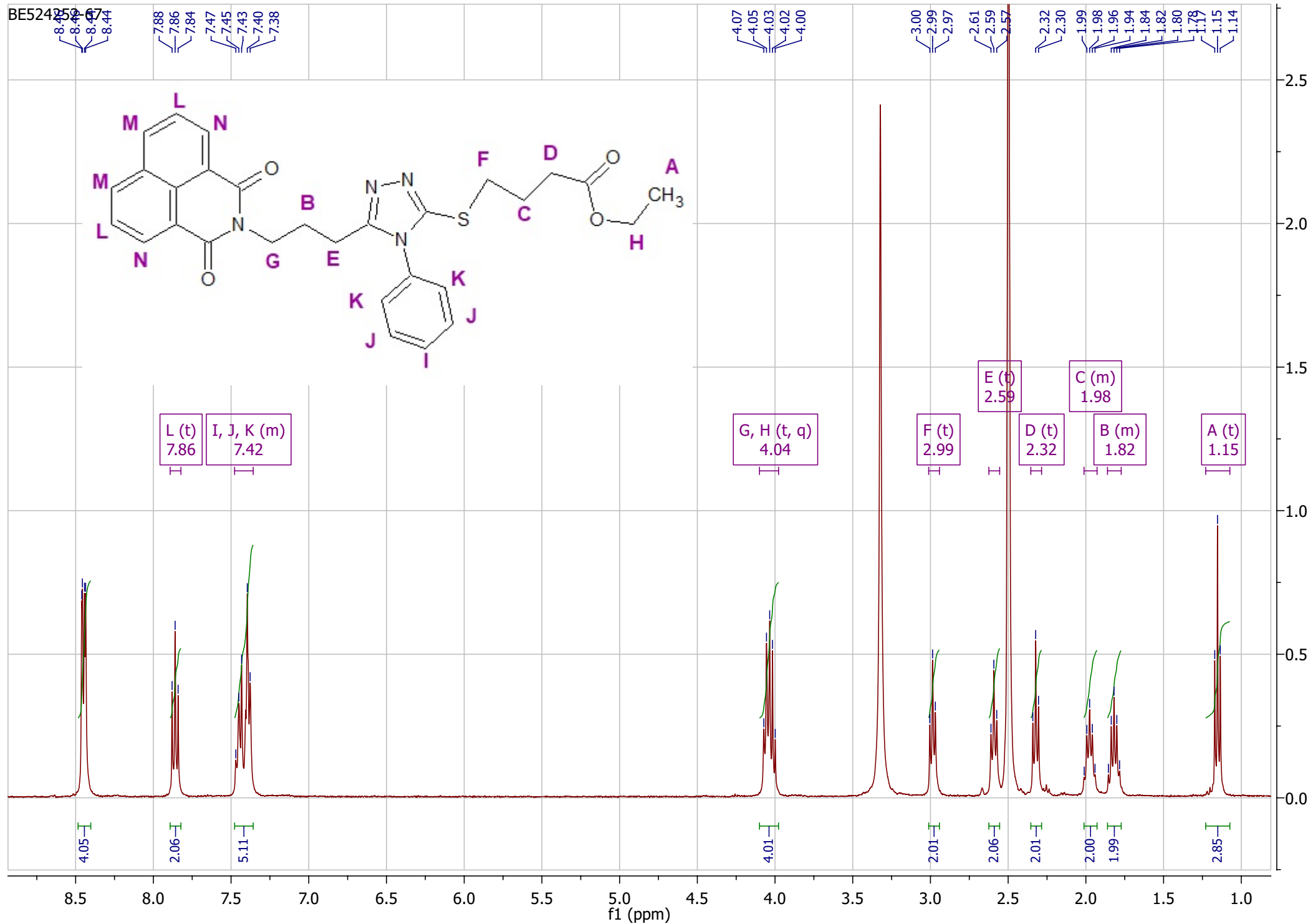

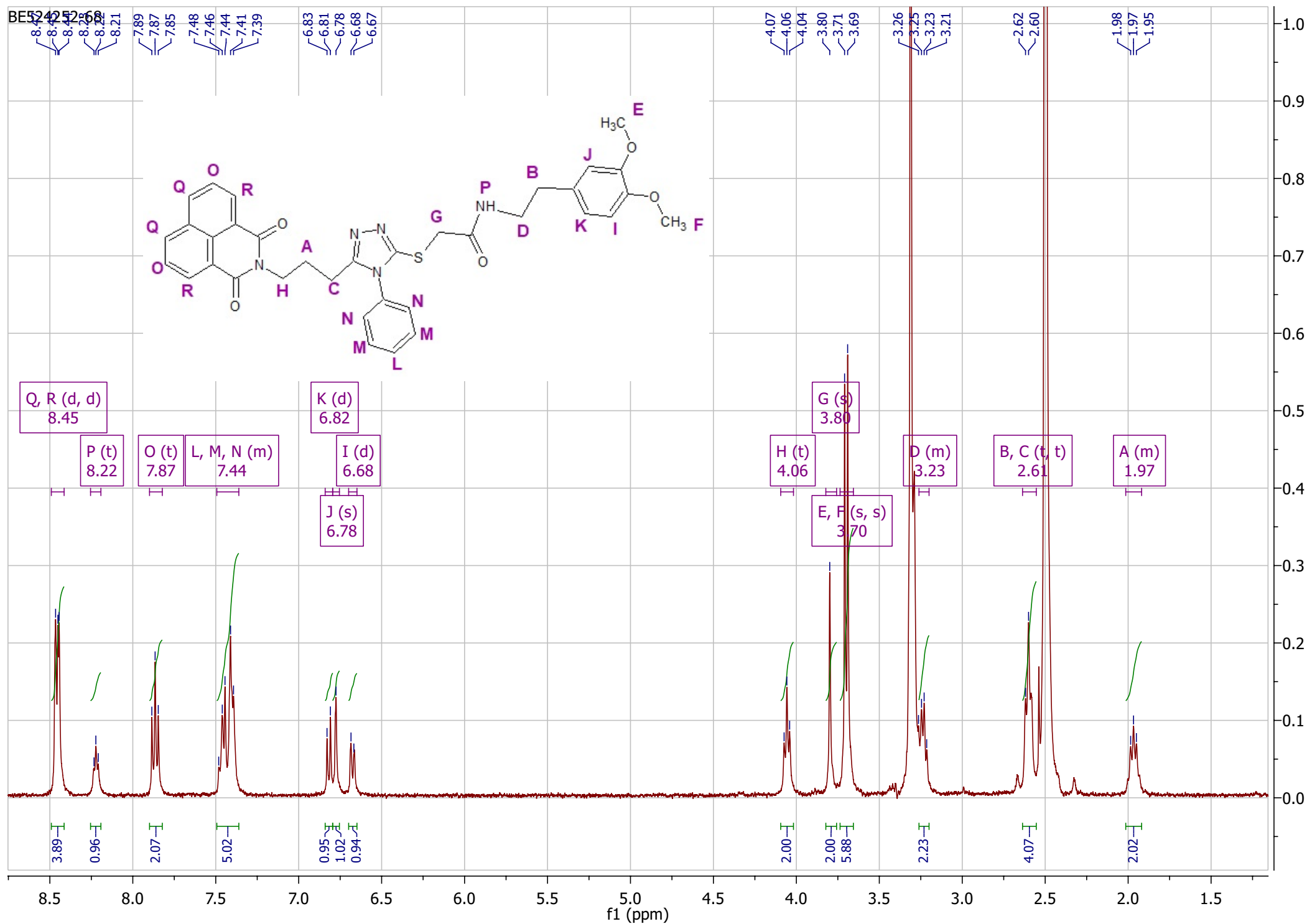

BE524252-69

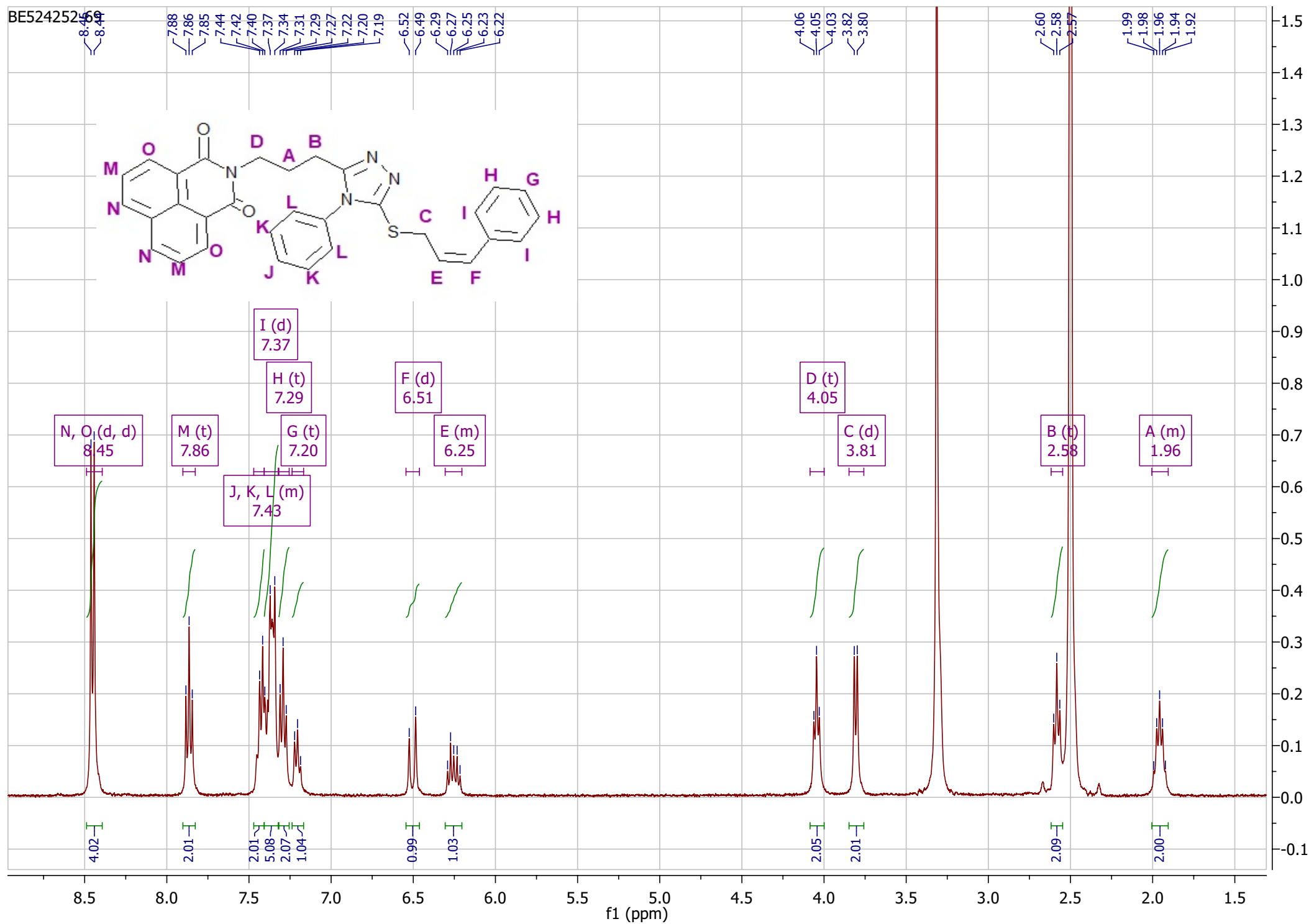

BE524252-71

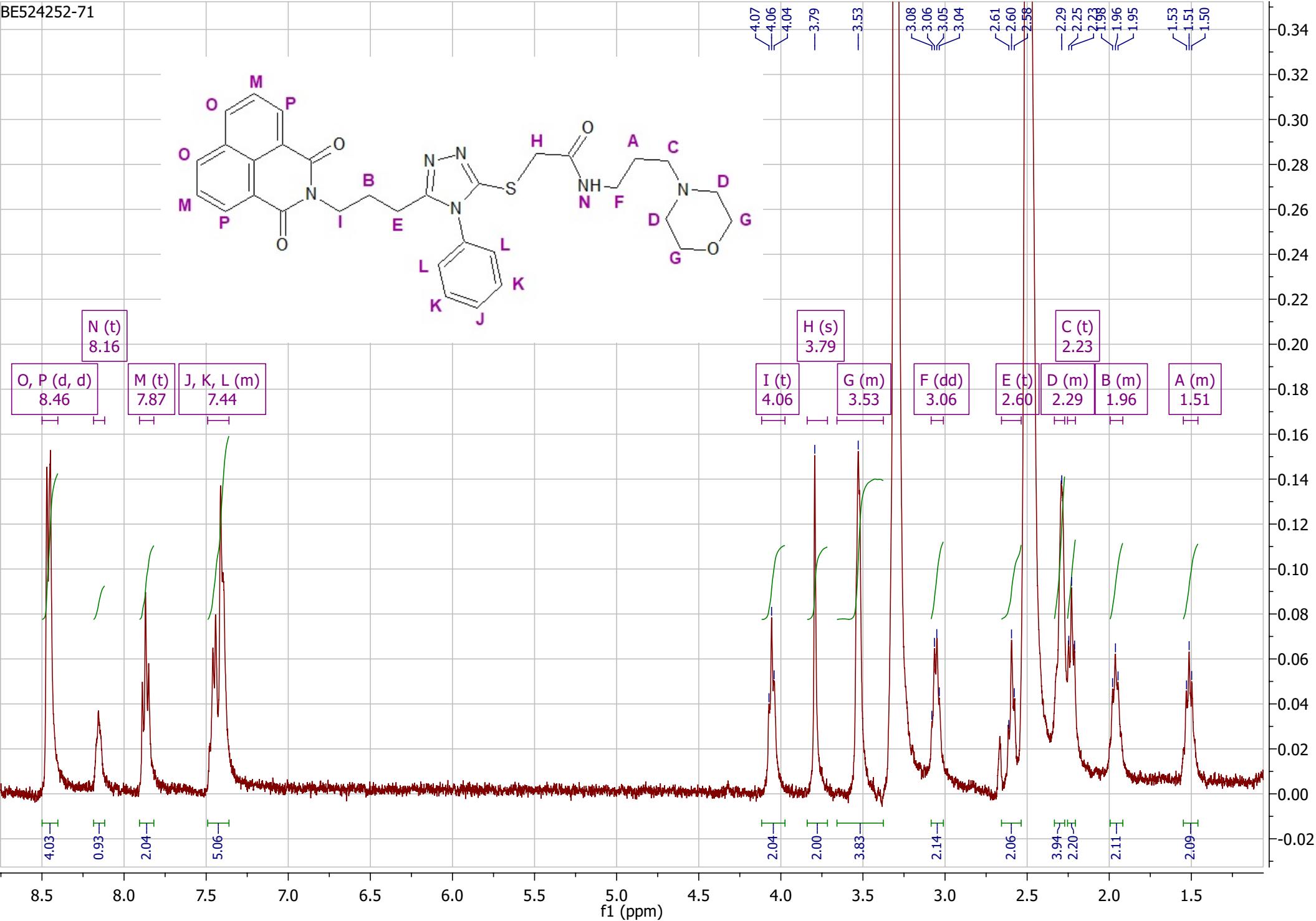

BE524252-72

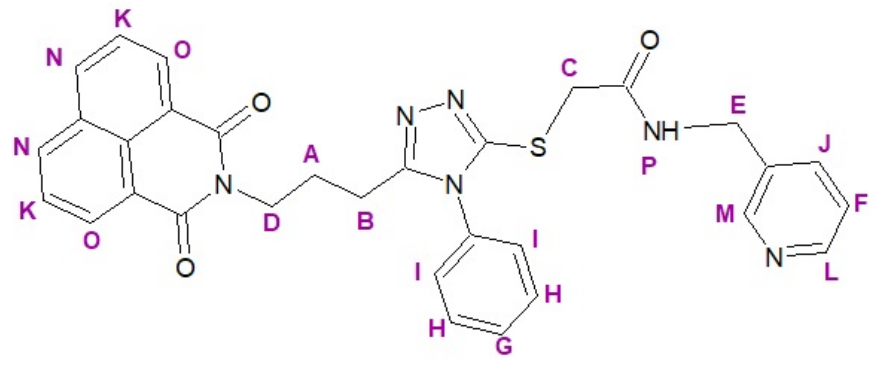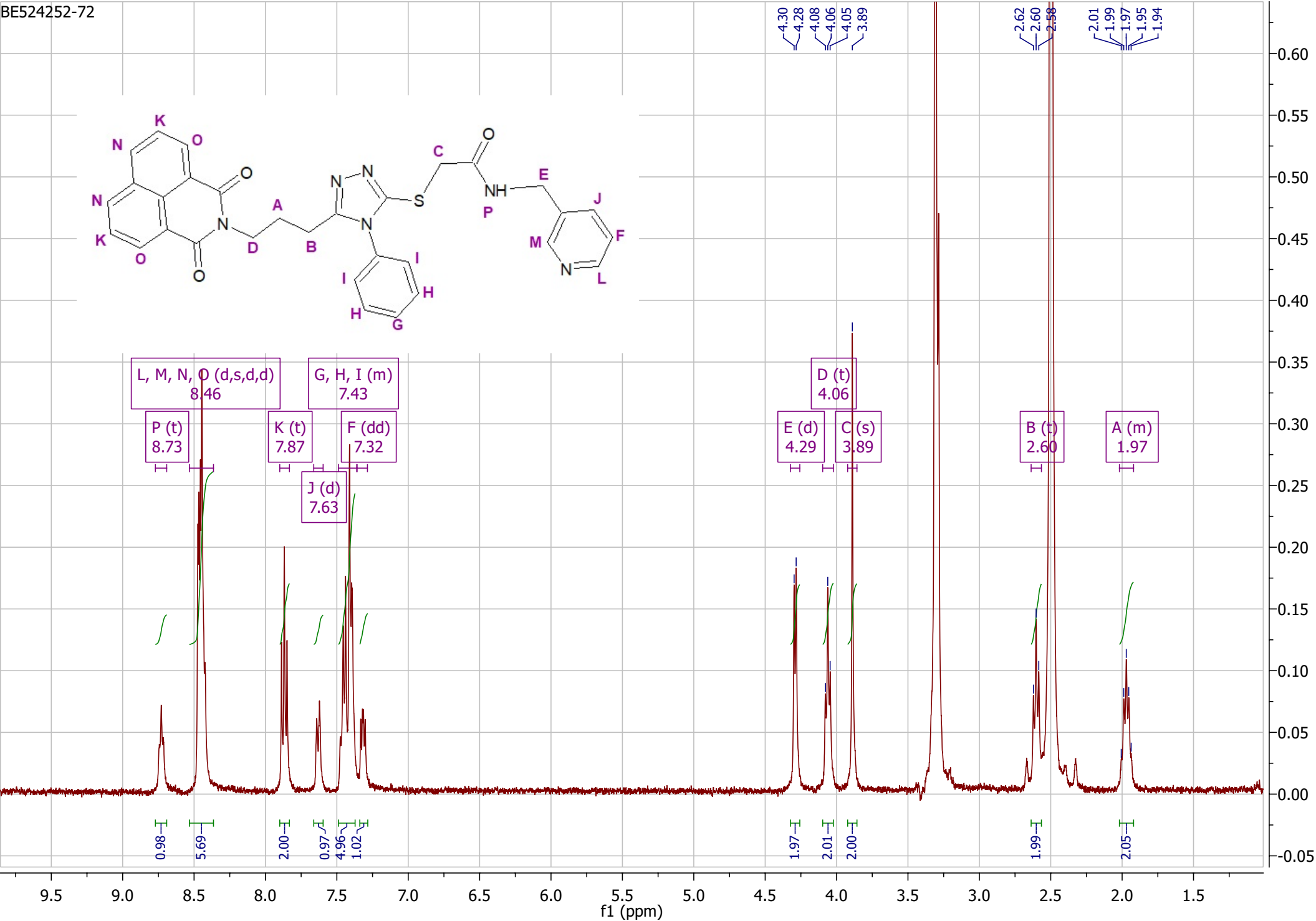

BE524252-60\_C13

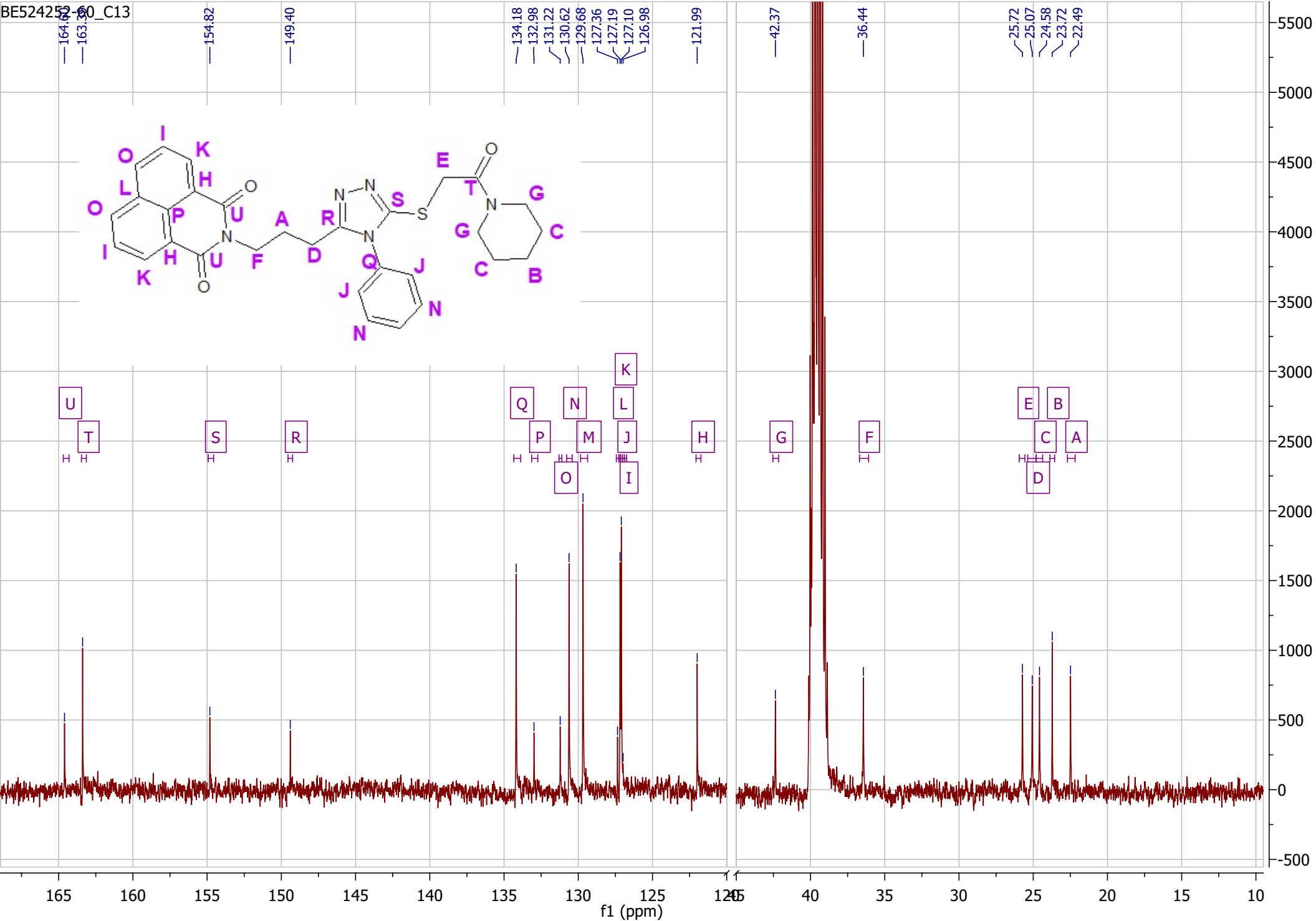

BE524252-63\_C13

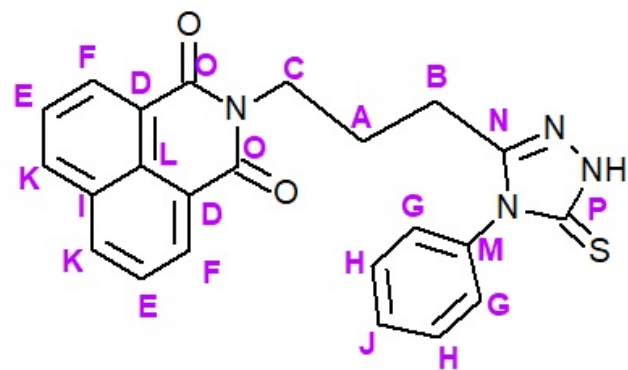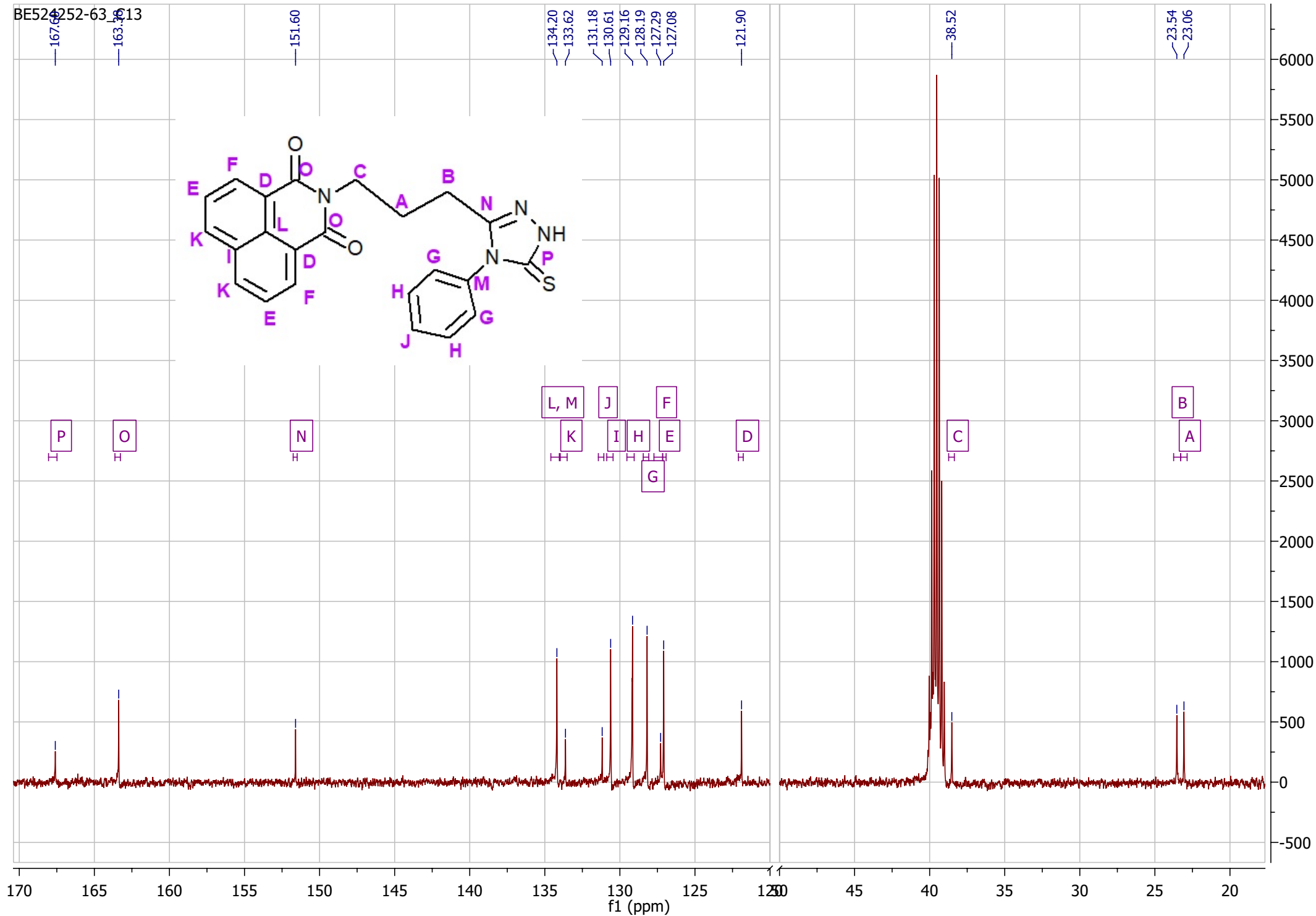

BE524252-67\_C13

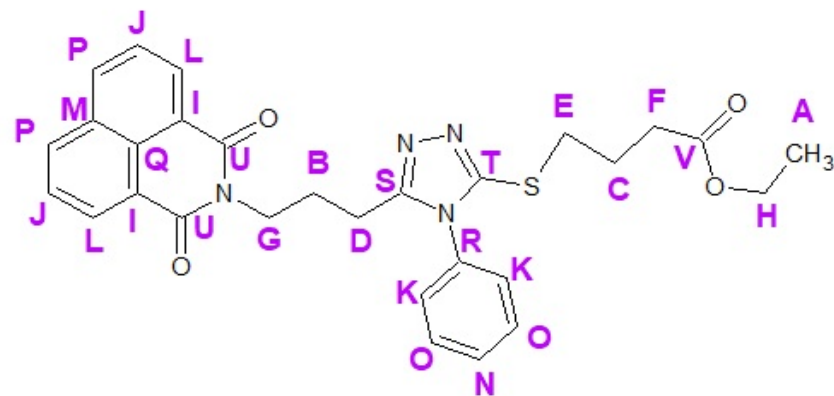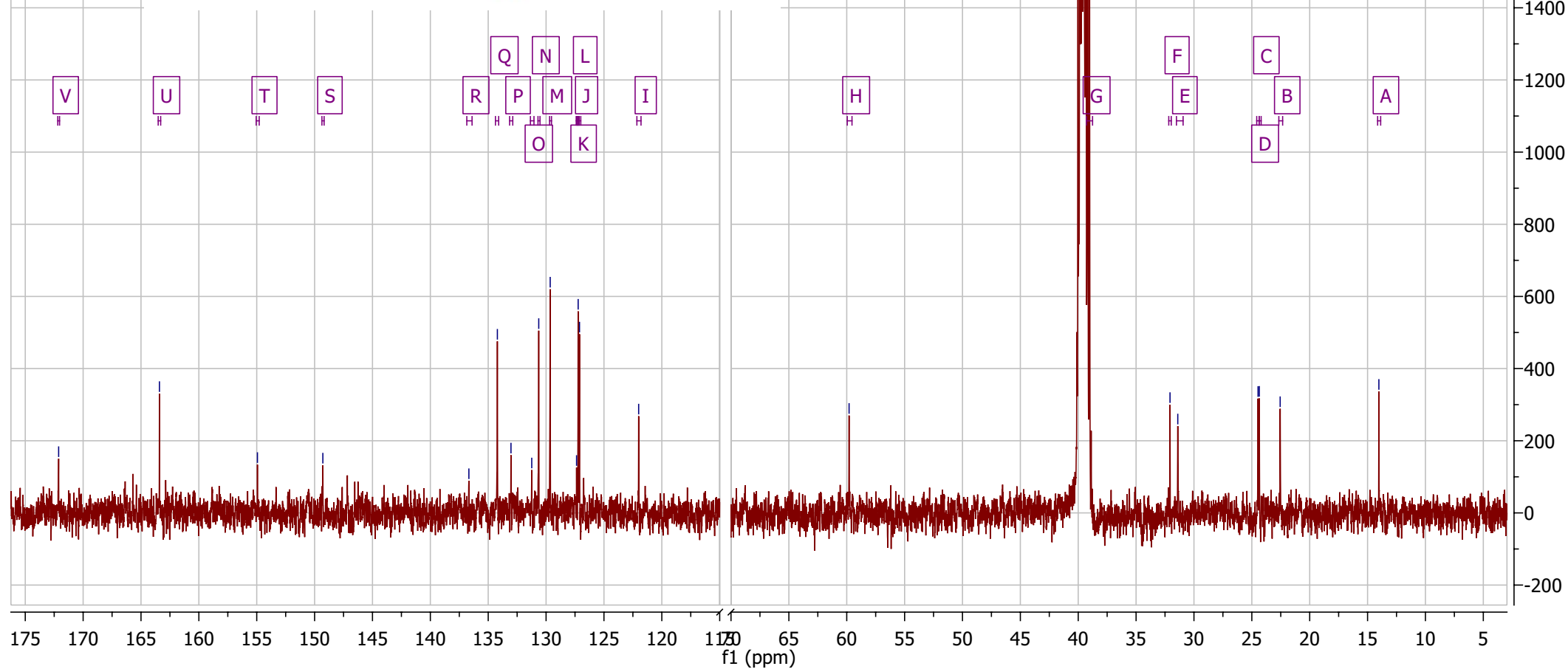

BE524252-68\_C13

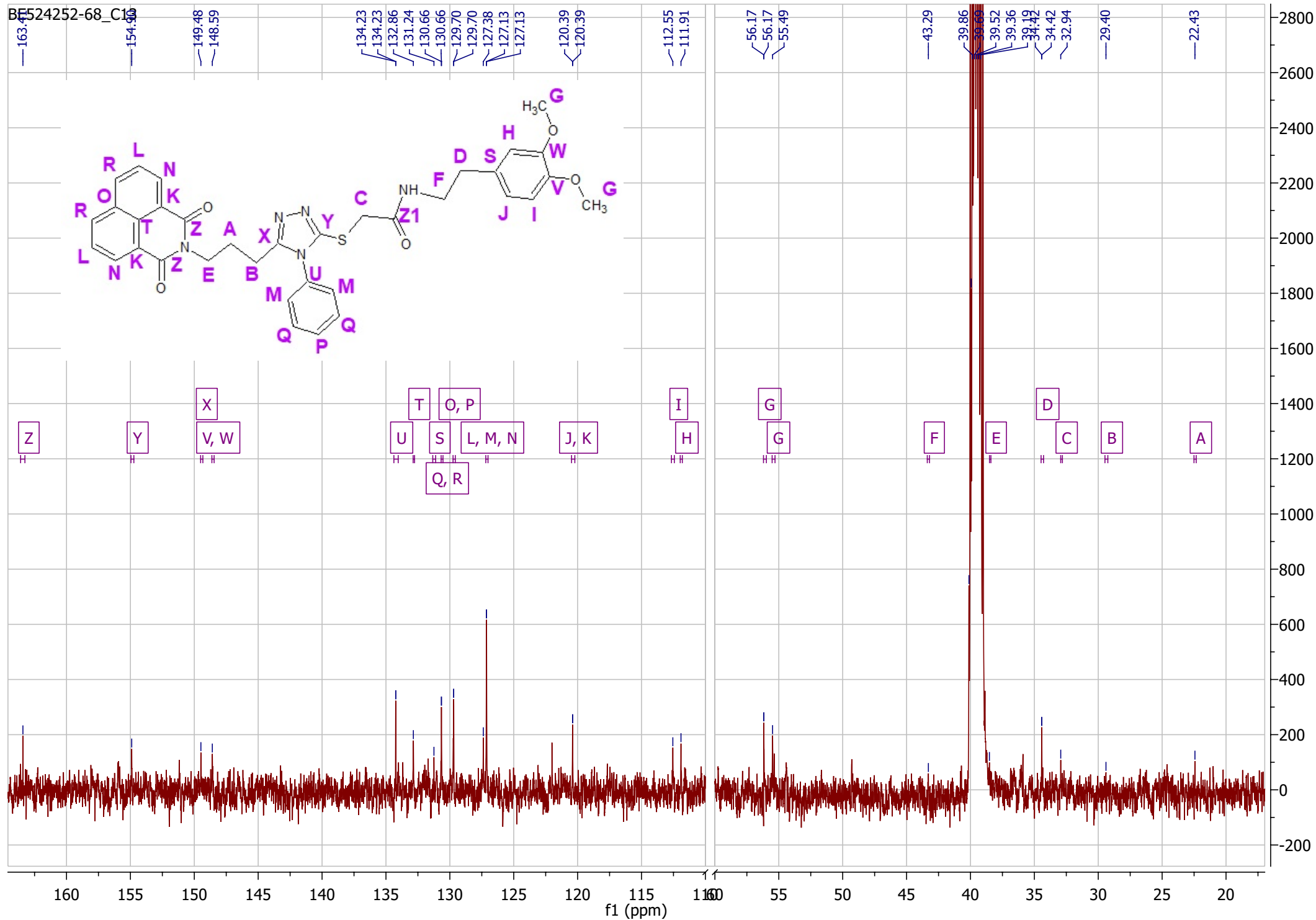

BE52425269\_C13

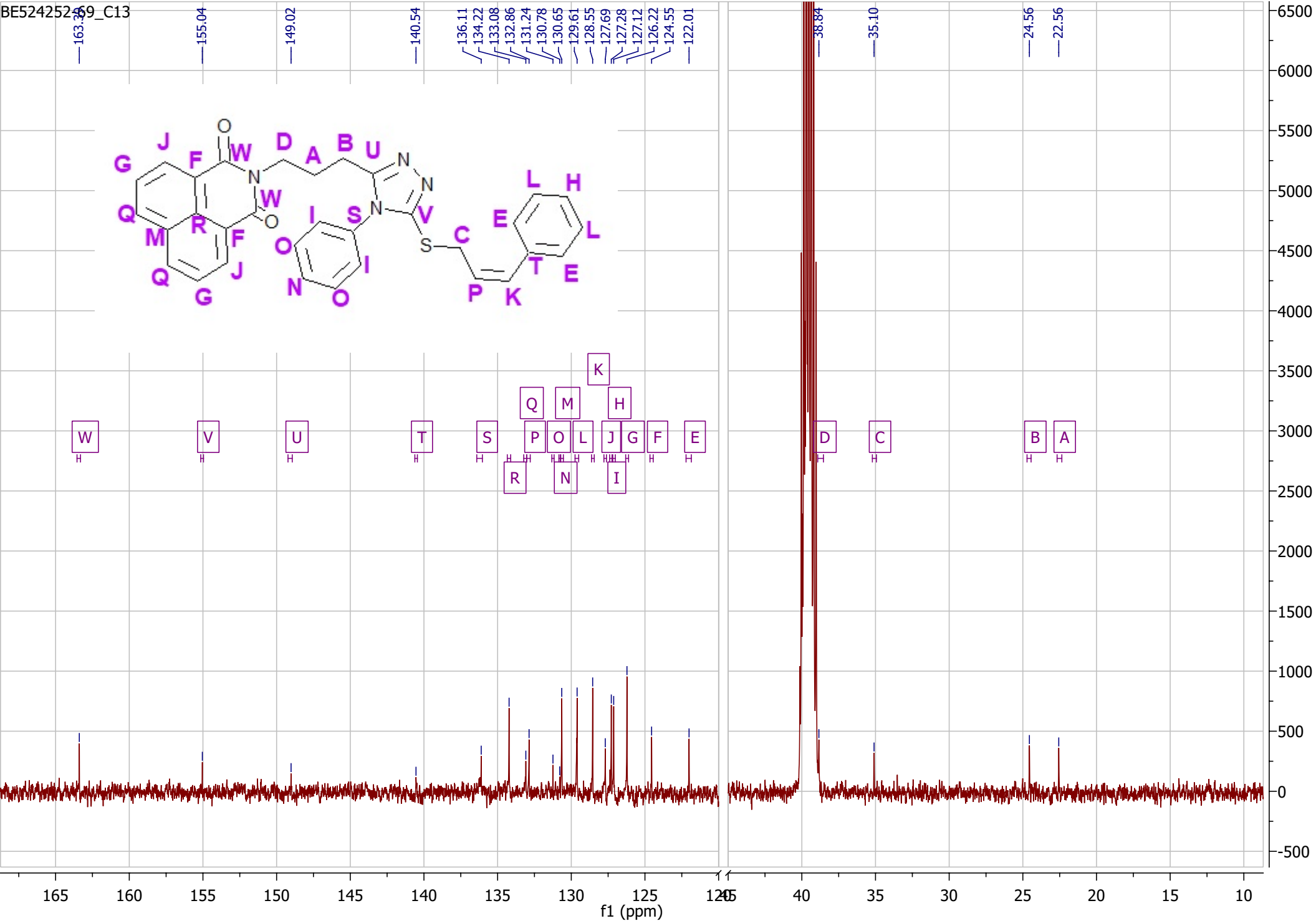

BE524252-70\_C13

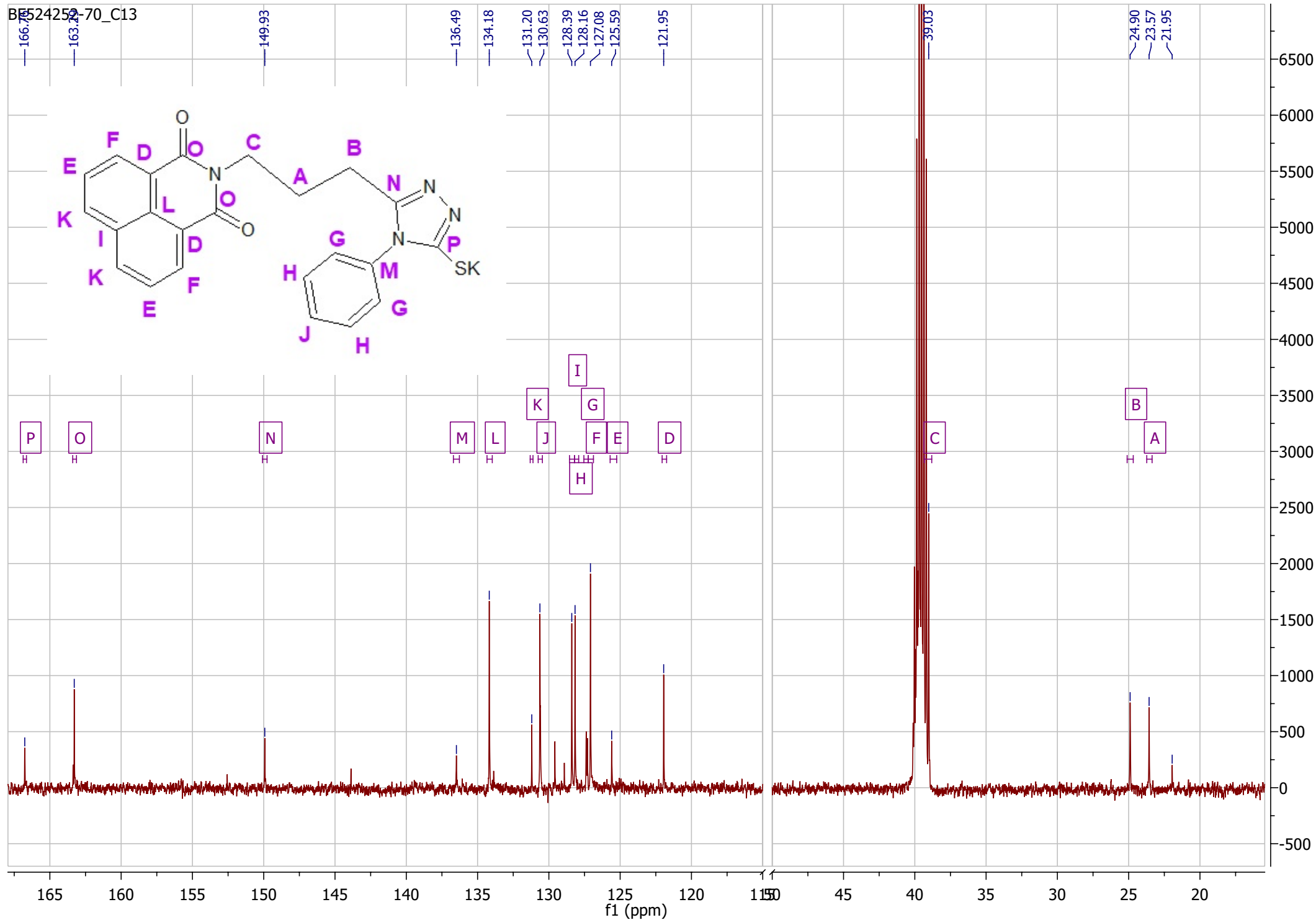

BE59425272\_C13

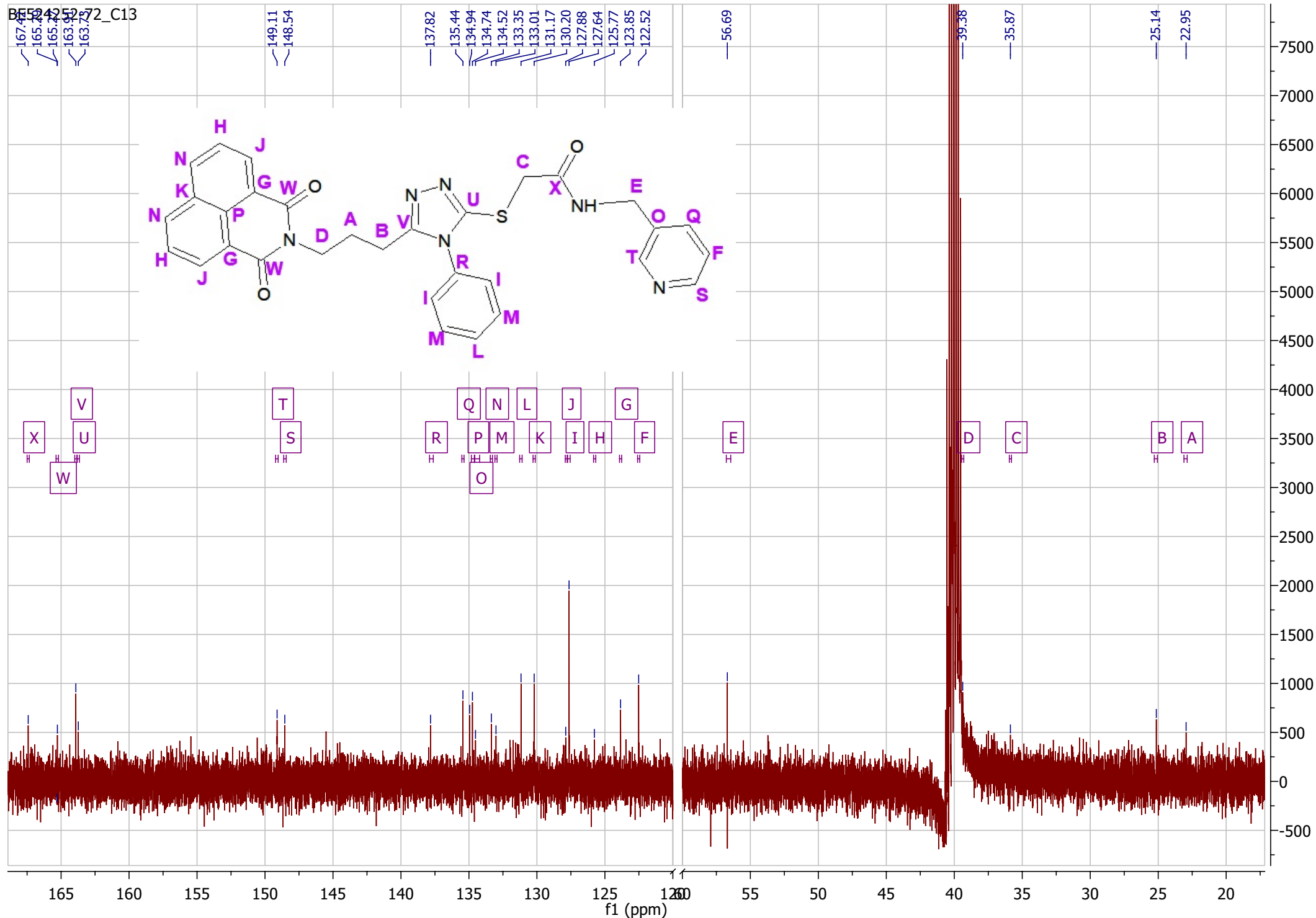

BE524252-71\_C13

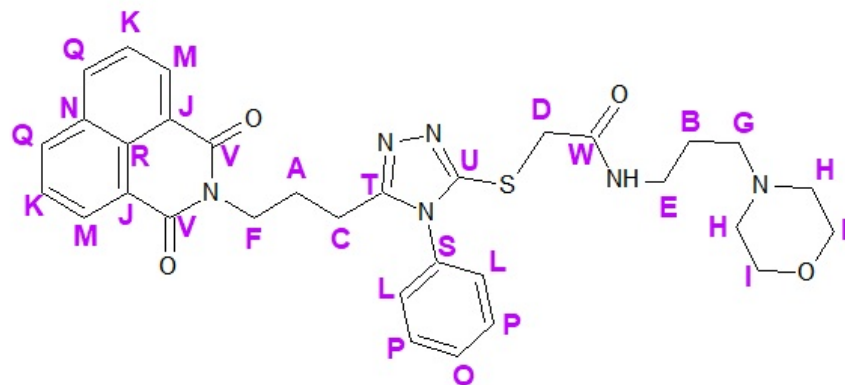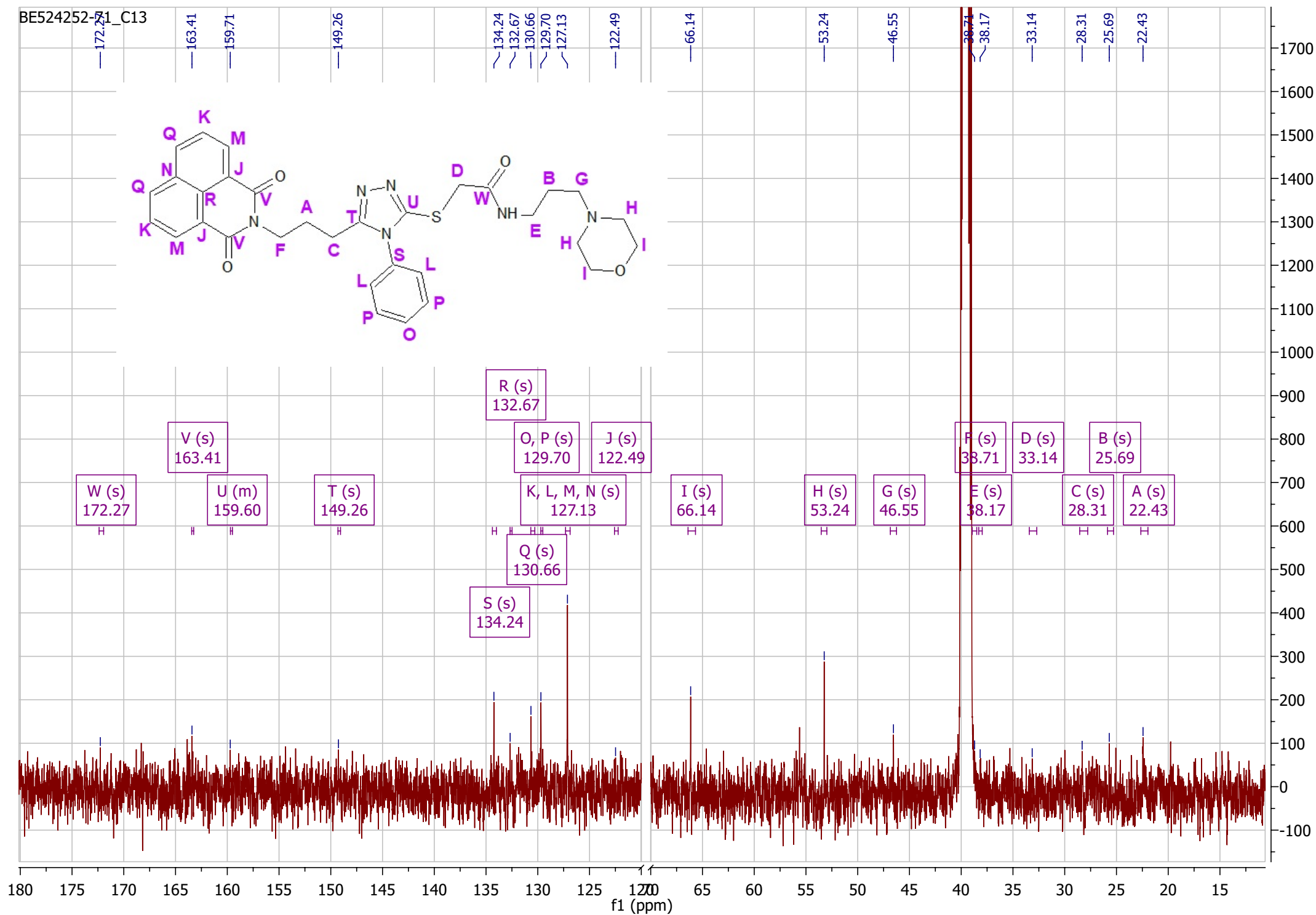

BE52425261\_C13

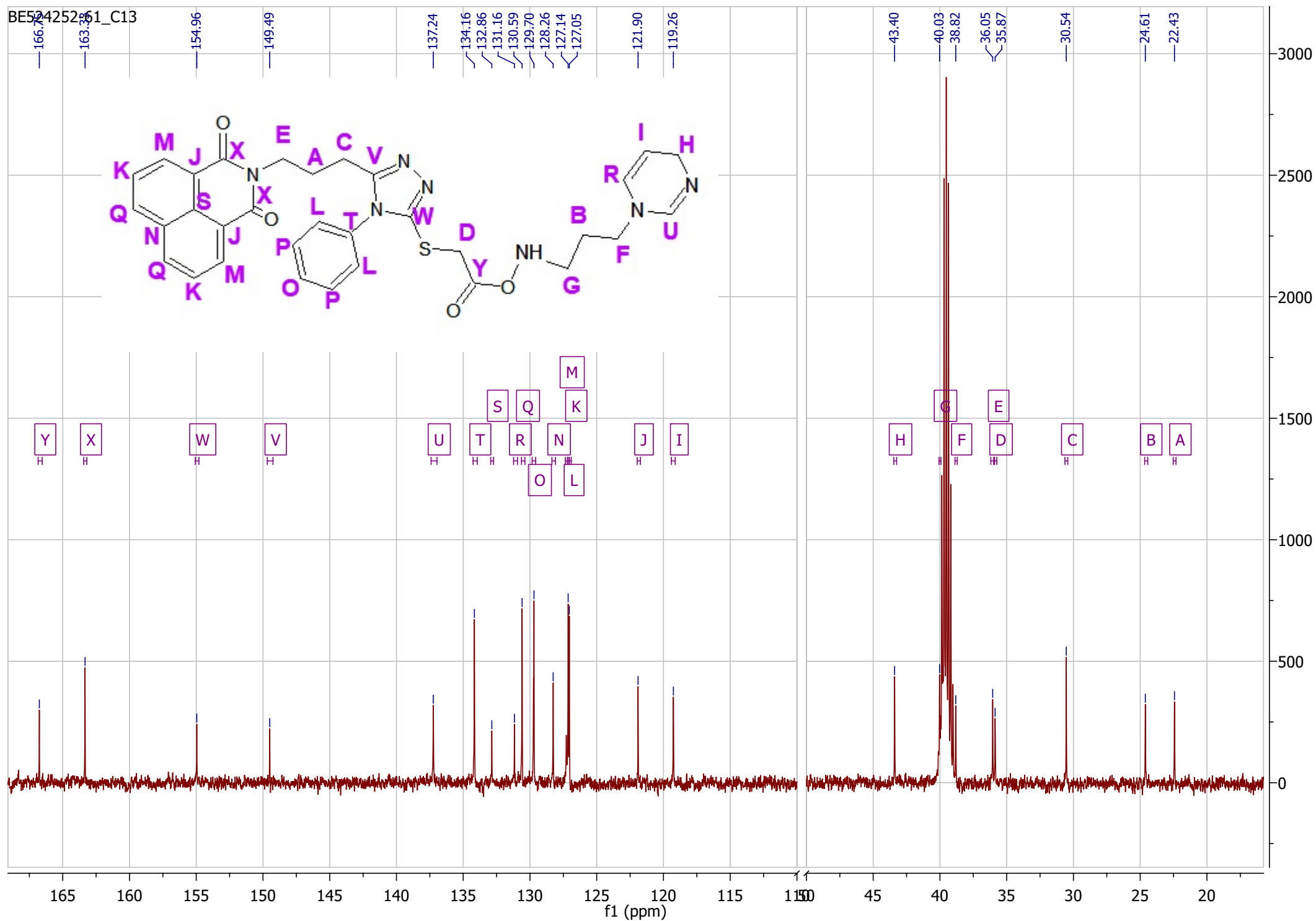

Supplement: Supporting Information — Additional supporting information can be found online in the Supporting Information section. File S1: NMR spectra (1H, 13C) of all synthesized compounds (PDF). [file 6115993.f1.pdf]
